# An integrated chromosome-scale genome assembly of the Masai giraffe (*Giraffa camelopardalis tippelskirchi*)

**DOI:** 10.1093/gigascience/giz090

**Published:** 2019-07-30

**Authors:** Marta Farré, Qiye Li, Iulia Darolti, Yang Zhou, Joana Damas, Anastasia A Proskuryakova, Anastasia I Kulemzina, Leona G Chemnick, Jaebum Kim, Oliver A Ryder, Jian Ma, Alexander S Graphodatsky, Guoije Zhang, Denis M Larkin, Harris A Lewin

**Affiliations:** 1 Department of Comparative Biomedical Sciences, Royal Veterinary College, University of London, London NW1 0TU, UK; 2 School of Biosciences, University of Kent, Canterbury CT2 7NJ, UK; 3 State Key Laboratory of Genetic Resources and Evolution, Kunming Institute of Zoology, Chinese Academy of Sciences, Kunming 650223, China; 4 China National Genebank, BGI-Shenzhen, Shenzhen 518083, China; 5 Department of Genetics, Evolution and Environment, University College London, London WC1E 6BT, UK; 6 Centre for Social Evolution, Department of Biology, Universitetsparken 15, University of Copenhagen, DK-2100 Copenhagen, Denmark; 7 The Genome Center, University of California, Davis, CA 95616, USA; 8 Institute of Molecular and Cellular Biology, SB RAS, Novosibirsk 630090, Russia; 9 Novosibirsk State University, Novosibirsk 630090, Russia; 10 San Diego Institute for Conservation Research, San Diego Zoo Global, Escondido, CA, USA; 11 Department of Biomedical Science and Engineering, Konkuk University, Seoul 05029, South Korea; 12 Computational Biology Department, School of Computer Science, Carnegie Mellon University, Pittsburgh, PA 15213, USA; 13 The Federal Research Center Institute of Cytology and Genetics, The Siberian Branch of the Russian Academy of Sciences (ICG SB RAS), Novosibirsk 630090, Russia; 14 Department of Evolution and Ecology, College of Biological Sciences, and the Department of Reproduction and Population Health, School of Veterinary Medicine, University of California, Davis, CA 95616, USA

**Keywords:** giraffe, *Giraffa camelopardalis tippelskirchi*, assembly, annotation, ruminant

## Abstract

**Background:**

The Masai giraffe (*Giraffa camelopardalis tippelskirchi*) is the largest-bodied giraffe and the world's tallest terrestrial animal. With its extreme size and height, the giraffe's unique anatomical and physiological adaptations have long been of interest to diverse research fields. Giraffes are also critical to ecosystems of sub-Saharan Africa, with their long neck serving as a conduit to food sources not shared by other herbivores. Although the genome of a Masai giraffe has been sequenced, the assembly was highly fragmented and suboptimal for genome analysis. Herein we report an improved giraffe genome assembly to facilitate evolutionary analysis of the giraffe and other ruminant genomes.

**Findings:**

Using SOAPdenovo2 and 170 Gbp of Illumina paired-end and mate-pair reads, we generated a 2.6-Gbp male Masai giraffe genome assembly, with a scaffold N50 of 3 Mbp. The incorporation of 114.6 Gbp of Chicago library sequencing data resulted in a HiRise SOAPdenovo + Chicago assembly with an N50 of 48 Mbp and containing 95% of expected genes according to BUSCO analysis. Using the Reference-Assisted Chromosome Assembly tool, we were able to order and orient scaffolds into 42 predicted chromosome fragments (PCFs). Using fluorescence in situ hybridization, we placed 153 cattle bacterial artificial chromosomes onto giraffe metaphase spreads to assess and assign the PCFs on 14 giraffe autosomes and the X chromosome resulting in the final assembly with an N50 of 177.94 Mbp. In this assembly, 21,621 protein-coding genes were identified using both *de novo* and homology-based predictions.

**Conclusions:**

We have produced the first chromosome-scale genome assembly for a Giraffidae species. This assembly provides a valuable resource for the study of artiodactyl evolution and for understanding the molecular basis of the unique adaptive traits of giraffes. In addition, the assembly will provide a powerful resource to assist conservation efforts of Masai giraffe, whose population size has declined by 52% in recent years.

## Background

**Figure 1:**
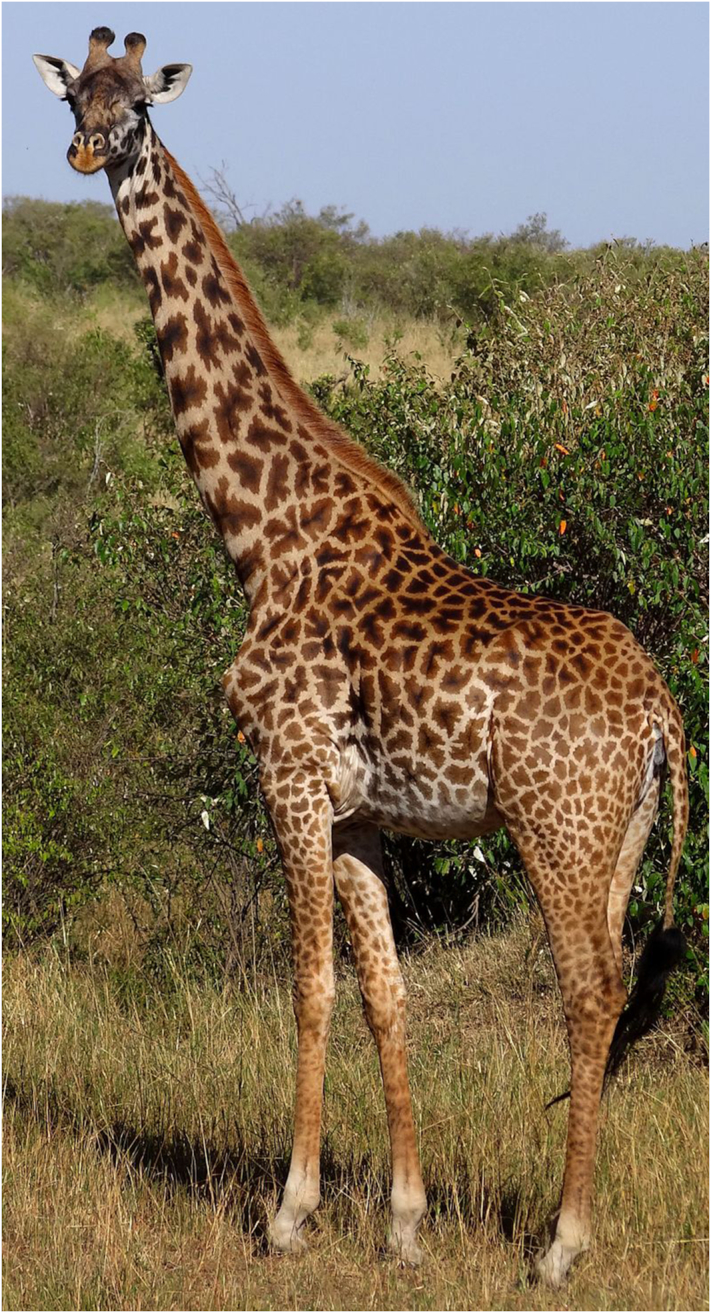
A representative adult female Masai giraffe (*Giraffa camelopardalis tippelskirchi*) in the Masai Mara national park, Kenya. Picture taken by Bjørn Christian Tørrissen, licence CC BY-SA 3.0.

Giraffes (*Giraffa*) are a genus of even-toed ungulate mammals comprising 4 species [1]. They are members of the family Giraffidae, which also includes the okapi (*Okapia johnstoni*). The Masai giraffe (also known as Kilimanjaro giraffe; *Giraffa camelopardalis tippelskirchi*; Fig. 1) is native to East Africa and distributed throughout Tanzania and Kenya [2]. Masai giraffes are not only the largest-bodied giraffes [3] but also the tallest terrestrial animals. Giraffes present several distinctive anatomical characteristics, such as their long neck and legs, horn-like ossicones, and coat patterns, which together with their unique cardiovascular and musculoskeletal adaptations have interested researchers in many fields [3–6].

The giraffe genome comprises 15 pairs of chromosomes (2n = 30) that are believed to have originated by multiple Robertsonian fusions from the pecoran ancestral karyotype (2n = 58) [7, 8]. In 2016, Agaba and colleagues [9] generated the first genome sequence of a female Masai giraffe and compared it with the genome sequence of an okapi. This study identified candidate genes and pathways involved in the giraffes’ unique skeletal and cardiovascular adaptations [9]. The reported genome was fragmented, which hinders its use for studies of overall genome architecture and evolution. Missing and fragmented genes also limit the utility of the assembly for study of the genetic basis of the giraffe's unique adaptations. Here we report a chromosome-scale assembly of a female Masai giraffe genome sequenced *de novo*. This assembly will facilitate studies of ruminant genome evolution and will be a powerful resource for further elucidation of the genetic basis for the giraffe's characteristic features. Furthermore, having another Masai giraffe genome sequence will assist conservation efforts for this species, whose population has declined by more than 52% in recent decades [2, 10].

## Data Description

### Library construction, sequencing, and filtering

Genomic DNA was extracted from a heart muscle sample OR1865 of a male Masai giraffe (Studbook no. 2336; Taxonomy ID: NCBI: txid439328) using the DNeasy Blood & Tissue Kit (QIAGEN, Valencia, CA, USA) according to the manufacturer's instructions. Isolated genomic DNA was then used to construct 12 sequencing libraries, 4 short-insert (170, 250, 500, and 800 bp) and 8 long-insert size (2, 5, 10, and 20 Kbp), following Illumina (San Diego, CA, USA) standard protocols. Using a whole-genome shotgun sequencing strategy on the Illumina HiSeq 2000 platform, we generated 296.23 Gbp of raw sequencing data with 100 bp or 50 bp paired-end sequencing for the short-insert or long-insert size libraries, respectively (Supplementary Table 1). To improve read quality, low-quality bases from both ends of the reads were trimmed and duplicated reads and those with more than 5% of uncalled (“N”) bases were removed. A total of 171.09 Gbp of filtered read data were used for genome assembly (Supplementary Table 1).

Two Chicago libraries were generated by Dovetail Genomics (Santa Cruz, CA, USA) as previously described [11]. Briefly, high-molecular-weight DNA was reconstituted into chromatin *in vitro*, chemically cross-linked, and digested by restriction enzymes. The resulting digestion overhangs were filled in with a biotinylated nucleotide, and the chromatin was incubated in a proximity-ligation reaction. The cross-links were then reversed and the DNA purified from chromatin. These libraries were sequenced in 1 flow-cell lane using the Illumina HiSeq 4000 platform, resulting in the generation of ∼385 million read pairs or 114.60 Gbp of sequence data (Supplementary Table 1).

### Evaluation of genome size

The Masai giraffe genome size was estimated by k-mer analysis. A k-mer refers to an artificial sequence division of K nucleotides iteratively from sequencing reads. A raw sequence read with L bp contains (L-K+1) different k-mers of length K bp. K-mer frequencies can be calculated from the genome sequence reads and typically follow a Poisson distribution when plotted against the sequence depth gradient. The genome size, G, can then be calculated from the formula G = K_num/K_depth, where the K_num is the total number of k-mers, and K_depth denotes the depth of coverage of the k-mer with the highest frequency. For giraffe, at K = 17, K_num was 75,710,429,964 and the K_depth was 30. Therefore, we estimated the genome size of *Giraffa camelopardalis tippelskirchi* to be 2.5 Gbp, comparable to the C-value of 2.7 and 2.9 reported for reticulated giraffe (*Giraffa camelopardalis reticulata*) [12]. All the filtered Illumina sequencing reads provided approximately 68.44× mean coverage of the genome, while the Chicago libraries’ reads presented an estimated genome coverage of 88.41×.

### Genome assembly

We applied SOAPdenovo version 2.04 (SOAPdenovo, RRID:SCR_010752) with default parameters to construct contigs and scaffolds as described previously [13]. All reads were aligned against each other to produce contigs that were further assembled in scaffolds using the paired-end information. The generated Masai giraffe genome assembly was 2.55 Gbp long, including 76.82 Mbp (3%) of unknown bases (“Ns”). The contig and scaffold N50 lengths were 21.78 Kbp and 3.00 Mbp, respectively (Table 1). To assess the assembly quality, approximately 90 Gbp (representing 35.6× genome coverage) high-quality, short-insert size reads were aligned to the SOAPdenovo assembly using BWA (BWA, RRID:SCR_010910), with parameters of -t 1 -I. A total of 98.9% reads could be mapped covering 98.9% of the assembly excluding gaps. Approximately 92% of these reads were properly paired, having an expected insert size associated with the libraries of origin.

**Table 1:** Assembly statistics of the *Giraffa camelopardalis tippelskirchi* genome

	ASM165123*	SOAPdenovo	SOAPdenovo + Chicago	SOAPdenovo + RACA	SOAPdenovo + Chicago + RACA	FINAL assembly
**Total length (Mbp)**	2,705.07	2,551.62	2,554.82	2,391.72	2,425.09	2,437.09
**N50 (Mbp)**	0.21	3.00	57.20	85.22	88.36	177.94
**No. scaffolds/predicted chromosome fragments (PCFs)**	513,177	739,028	735,884	47	42	24
**Gap sequence (%)**	3.48	3.01	3.13	3.06	3.22	3.69
**No. input scaffolds/PCFs broken**	—	—	54	35	16	0

^*^Agaba et al. 2016.

To increase the contiguity of the assembly, we used the HiRise2.1 scaffolder [11] and sequence information from the Chicago libraries and SOAPdenovo assembly as inputs. The SOAPdenovo + Chicago assembly introduced a total of 56 breaks in 54 SOAPdenovo scaffolds and formed 3,200 new scaffold joints, resulting in an increased scaffold N50 length of 57.20 Mbp (Table 1).

#### Evaluation of the SOAPdenovo genome assembly and PCR verification of putatively chimeric scaffolds

To identify putatively chimeric scaffolds, we used the Masai giraffe SOAPdenovo genome assembly to obtain predicted chromosome fragments (PCFs) using Reference-Assisted Chromosome Assembly (RACA) software [14]. The RACA tool uses a combination of comparative information and sequencing data to order and orient scaffolds of target species and generate PCFs. The cattle (*Bos taurus*, bosTau6) and human (*Homo sapiens*, hg19) genome assemblies were used as a reference and outgroup, respectively, and all Illumina paired-end and mate-pair libraries were included in the RACA assembly. The read libraries were aligned to the SOAPdenovo scaffolds using Bowtie2 (Bowtie, RRID:SCR_005476) [15]. The cattle-giraffe and cattle-human pairwise alignments were performed using lastZ and UCSC Kent utilities [16], as previously described [14, 17]. The RACA software was used at a minimum resolution of 150 Kbp for syntenic fragment (SF) detection. Only SOAPdenovo scaffolds >10 Kbp were used as input for RACA, comprising 95% of the assembly length.

After an initial run of RACA with default parameters, we tested the structure of 32 of 41 (76%) RACA-split SF adjacencies corresponding to 40 SOAPdenovo scaffolds flagged as putatively chimeric. Chimerism was evaluated using PCR amplification of Masai giraffe DNA with primers that flank the RACA-defined split of SF joint boundaries (Supplementary Table 2 and Supplementary Table 3). Because we were only able to test 76% of the putatively chimeric SOAPdenovo scaffolds, we mapped short- and long-insert size read libraries to the SOAPdenovo assembly to establish a minimum physical coverage of reads that mapped across the SF joint intervals, following previous publications [18]. By comparing the PCR results and the read mapping coverage, we established 158x as the minimum physical coverage that allowed differentiation of scaffolds that were likely to be chimeric from those that were likely to be authentic (Supplementary Table 2). This threshold was used to update the parameters of a second round of RACA (stage 2 RACA), which resulted in the generation of 47 PCFs, of which 13 were homologous to complete cattle chromosomes. The stage 2 RACA assembly had an N50 length of 85.22 Mbp. This assembly comprised 1,283 SOAPdenovo scaffolds, representing 93% of the original SOAPdenovo assembly, of which 33 were split by RACA, and 2 were manually split as they had been shown to be chimeric by PCR (Table 1). These results indicate the power of comparative information for improving assembly contiguity and for identifying problematic regions in *de novo* assemblies.

#### Evaluation of the HiRise SOAPdenovo + Chicago assembly

More than 94% of the joints introduced in the SOAPdenovo + Chicago assembly were concordant with the RACA assembly, 4% were inconsistent between the 2 assemblies, and 1% represented extra adjacencies with intervening scaffolds located at the ends of PCFs. Among the 54 SOAPdenovo scaffolds broken in the SOAPdenovo + Chicago assembly, 26 were also broken in the RACA assembly. Among the remaining 28 scaffolds, 5 were not included in PCFs because they were under the 150-Kbp SF resolution set in the RACA tool; 16 were broken in the Chicago assembly, with 1 of the fragments below SF resolution, and 7 scaffolds were broken in the SOAPdenovo + Chicago assembly and intact in the RACA assembly (SOAPdenovo scaffolds 82, 813, 816, 849, 906, 940, and 995). Additionally, among the 16 SOAPdenovo scaffolds PCR-verified to be chimeric, 13 were also broken in the SOAPdenovo + Chicago assembly. The remaining 3 chimeric joints, within SOAPdenovo scaffolds 181, 267, and 696, were manually split in the SOAPdenovo + Chicago assembly (scaffolds Sc_7219; HRSCAF = 8,761 and Sc_732 785; HRSCAF = 735,706). The final SOAPdenovo + Chicago genome assembly comprises 2.55 Gbp and has an N50 length of 57.20 Mbp (Table 1).

Comparison to cattle chromosomes identified 5 chromosomal fusions in the giraffe SOAPdenovo + Chicago assembly. Two of those fusions (cattle chromosomes BTA1/BTA28 and BTA26/BTA28) were previously detected using cytogenetic approaches, and both locate on giraffe chromosome 2 [7, 8]. Finally, we ran RACA using the SOAPdenovo + Chicago scaffolds and cattle (bosTau6) and human (hg19) genomes as reference and outgroup, respectively. RACA produced 42 PCFs (Table 1), 20 of them representing complete cattle chromosomes, a substantial improvement over the SOAPdenovo + RACA assembly.

#### Evaluation of SOAPdenovo + Chicago + RACA assembly and scaffold placement into chromosomes using fluorescence *in situ* hybridization

**Figure 2:**
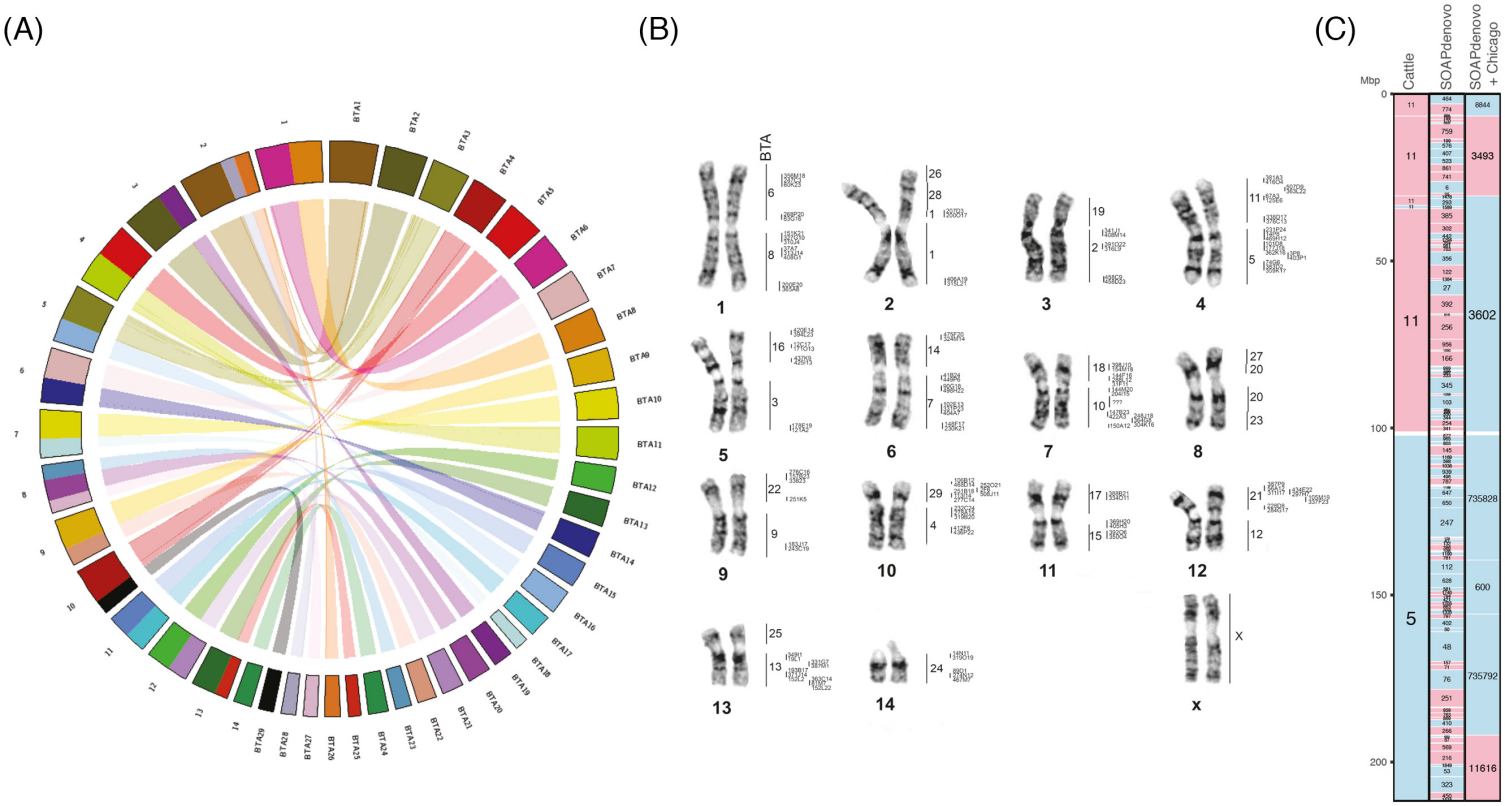
Syntenic relationships between giraffe and cattle genomes. (A) Circos plot showing syntenic relationships between cattle autosomes (labeled as BTA) and giraffe chromosomes. Chromosomes are colored based on cattle homologies. Ribbons inside the plot show syntenic relationships, while lines inside each ribbon indicate inversions. (B) Placement of cattle BACs onto the giraffe karyotype. The first column of numbers on the right of each pair of giraffe chromosomes corresponds to cattle (BTA) chromosomes, while the second column locates the cattle BAC IDs hybridized to giraffe chromosomes. (C) Giraffe chromosome 14 from the final assembly (Table1) showing homologous synteny blocks (HSBs) between giraffe and cattle. SOAPdenovo and SOAPdenovo + Chicago scaffolds are also displayed. Blue blocks indicate positive (+) orientation of tracks compared with the giraffe chromosome, while red blocks indicate negative (−) orientation. Numbers inside each block represent cattle chromosomes or giraffe scaffold IDs. BTA: *Bos taurus*, cattle. Images of all giraffe chromosomes can be found in Supplementary Fig. 1.

To assess and map the SOAPdenovo + Chicago + RACA PCFs onto giraffe chromosomes, we performed fluorescence *in situ* hybridization (FISH) of cattle bacterial artificial chromosomes (BACs) from the CHORI-240 library [19] with giraffe metaphase spreads (Fig. 2) following previous publications [20]. Briefly, giraffe fibroblast cells were incubated at 37°C and 5% CO_2_ in Alpha MEM (Gibco, USA) supplemented with 15% fetal bovine serum (Gibco, USA), 5% AmnioMAX-II (Gibco, USA), and antibiotics (ampicillin 100 μg/ml, penicillin 100 μg/ml, amphotericin B 2.5 μg/ml). Metaphases were obtained by adding colcemid (0.02 mg/ml) and EtBr (1.5 mg/ml) to actively dividing cultures. Hypotonic treatment was performed with KCl (3 mM) and sodium citrate (0.7 mM) for 20 min at 37°C and followed by fixation with 3:1 methanol-glacial acetic acid fixative. BAC DNA was isolated using a plasmid DNA isolation kit (Biosilica, Novosibirsk, Russia) and amplified using whole-genome amplification (GenomePlex Whole Genome Amplification Kit; Sigma, USA). Labeling of BAC DNA was performed using the GenomePlex WGA Reamplification Kit (Sigma, USA) by incorporating biotin-16-dUTP (Roche, USA) or digoxigenin-dUTP (Roche, USA). Two-color FISH experiments on G-banded metaphase chromosomes were performed as described previously [20].

BAC clone coordinates for cattle (bosTau6) assembly were downloaded from NCBI CloneDB [21] and converted to coordinates in the giraffe SOAPdenovo + Chicago + RACA PCFs using the UCSC Genome Browser LiftOver tool [22]. A total of 153 BACs were successfully mapped to the giraffe assembly and retained for the following analysis. To evaluate the 146 scaffold joints introduced by RACA, a reliability score was further calculated considering 4 components: (i) the relative positions of the BACs in giraffe metaphase spreads compared to the PCFs (Fig. 2), (ii) if the joint was supported by sequence reads from Chicago libraries, (iii) physical coverage of Illumina paired-end reads, and (iv) comparative syntenic information. Different weights were given to each component of the score, ranging from 10% for the comparative syntenic information to 40% for the physical map using BAC data (Supplementary Table 4). Only those joints with a reliability score >30% were considered authentic, indicating that at least FISH or Chicago library read support was present. More than 89% (*N* = 130) of the adjacencies had FISH and/or Chicago support, while 6 (4%) adjacencies had syntenic support only (Supplementary Fig. 1). The final genome assembly comprised PCFs placed on 14 giraffe autosomes and 10 chromosome X fragments (Table 1). Because chromosome X in Cetartiodactyls (including giraffe, cattle, and pigs) has been highly rearranged during evolution [20], tools such as RACA, which use a reference-assisted assembly approach, will have limited success in increasing the contiguity of the assembly of sex chromosomes in the Cetartiodactyl clade.

#### Completeness evaluation of genome assemblies using BUSCO

**Figure 3:**
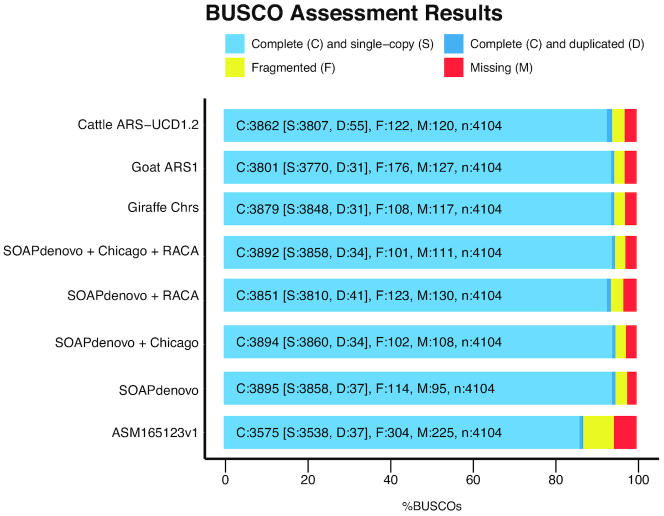
Benchmarking of genome completeness for the 4 giraffe assemblies using BUSCO. The BUSCO data set of the mammalia_odb9 including 4,104 genes was used to assess the completeness of the 4 giraffe genome assemblies, as well as the previously published giraffe genome (ASM165123v1 [9]). The newly released cattle (ARS−UCD1.2, GCA_0 022 63795.2) and goat (ARS1, GCA_0 017 04415.1) assemblies are included for comparison.

We evaluated genome completeness using the Benchmarking Universal Single-Copy Orthologs (BUSCO, RRID:SCR_015008; version 3.0) software [23]. Although comparing BUSCO results on different versions of genome assemblies might be inappropriate due to differences in parameter estimations [24], we found a high agreement between genome assemblies, with only 34 BUSCO single-copy genes present in the SOAPdenovo assembly reported missing in the final assembly, while 42 BUSCO genes reported as fragmented and an additional 14 reported as missing in the SOAPdenovo assembly were labeled as complete in the final assembly. Overall, approximately 95% of the core mammalian gene set was complete in the SOAPdenovo and SOAPdenovo + Chicago assemblies; SOAPdenovo + RACA included 94% of the mammalian gene set, while the final chromosome-level assembly contained 95% complete BUSCO genes, similar to other reference-quality ruminant assemblies (94% for cattle ARS-UCD1.2 and goat ARS1). In comparison, the Masai giraffe genome assembly reported by Agaba and colleagues [9] included 87% of BUSCO genes (Fig. 3). These results show that the genome assemblies we generated are of high completeness and accuracy, as well as a significant improvement over the genome assembly currently available for Masai giraffe.

### Genome annotation

To annotate transposable elements (TEs) in the Masai giraffe genome, we started by predicting TEs by homology to RepBase sequences using RepeatProteinMask and RepeatMasker (RepeatMasker, RRID:SCR_012954) [25] with default parameters. Results from both types of software were combined to produce a nonredundant final set of TEs. Approximately 40% of the Masai giraffe's genome comprises TEs, with LINEs being the most frequent group (24%, Supplementary Table 6).

The remainder of the SOAPdenovo genome assembly was annotated using both homology-based and *de novo* methods. For the homology-based prediction, human, mouse, cow, and horse proteins were downloaded from Ensembl (Ensembl, RRID:SCR_002344), release 64, and mapped onto the genome using tblastn. The homologous genome sequences were aligned against the matching proteins using GeneWise (GeneWise, RRID:SCR_015054) [26] to define gene models. For *de novo* prediction, Augustus (Augustus: Gene Prediction, RRID:SCR_008417) [27], GENSCAN (GENSCAN, RRID:SCR_012902) [28], and SNAP (SNAP, RRID:SCR_007936) [29] were applied to predict coding genes as described in Zhang et al. [30]. Finally, homology-based and *de novo* derived gene sets were merged to form a comprehensive and nonredundant reference gene set using GLEAN [31]. We obtained a reference gene set that contained 21,621 genes (Supplementary Table 7).

To assign functions to the newly annotated genes in the Masai giraffe genome, we aligned them to SwissProt database using blastp with an (E)-value cutoff of 1 e^−5^. A total of 18,910 genes (87.46% of the total annotated genes) had a Swissprot match. Publicly available databases, including Pfam (Pfam, RRID:SCR_004726), PRINTS (PRINTS, RRID:SCR_003412), PROSITE (PROSITE, RRID:SCR_003457), ProDom (ProDom, RRID:SCR_006969), and SMART (SMART, RRID:SCR_005026), were used to annotate motifs and domains in the gene sequences using InterPro (InterPro, RRID:SCR_006695), producing a total of 16,137 genes annotated with domain information (74.64%). By searching the KEGG database using a best hit for each gene, 9,087 genes were mapped to a known pathway (42.03% of the genes). Finally, we assigned a gene ontology term to 12,263 genes, representing 56.72% of the full gene set. Overall, 18,955 genes (87.67%) had at least 1 functional annotation (Supplementary Table 8).

### Genome evolution

**Figure 4:**
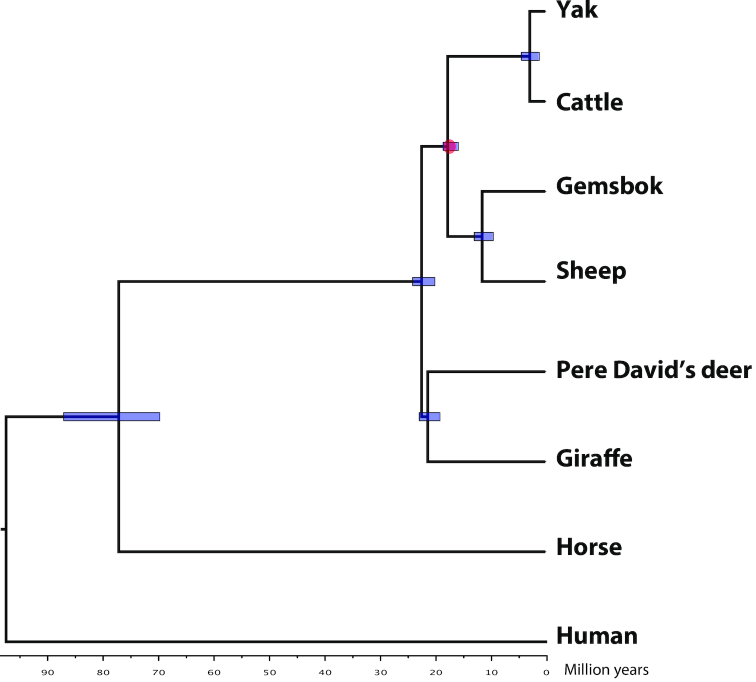
Phylogenetic relationships of the giraffe. Phylogenetic tree constructed with orthologous genes. Divergence times were extracted from the TimeTree database for calibration. Blue bars indicate the estimated divergence times in millions of years, and red circle indicates the calibration time.

The position of the Giraffidae family in the Ruminantia has been highly debated, with some studies using mitochondrial DNA or SNPchip data suggesting that Giraffidae are an outgroup to Bovidae and Cervidae [32, 33], while palaeontological and biochemical evidence suggested that Giraffidae and Cervidae are sister taxa [34, 35]. To shed light on the giraffe phylogeny, we first used the TreeFam methodology [36] to define gene families in 8 mammalian genomes (cattle, sheep, gemsbok, yak, giraffe, Pere David's deer, horse, and human) using newly defined or available gene annotations. We applied the same pipeline and parameters as described by Kim et al. [37]. A total of 16,148 gene families, of which 1,327 are single-copy orthologous families, were obtained. Concatenated protein sequence alignments of single-copy orthologous families were used as input for building the tree, with the JTT+gamma model, using PhyML v3.3 (PhyML, RRID:SCR_014629) [38]. Branch reliability was assessed by 1,000 bootstrap replicates. Finally, PAML mcmctree [39] was used to determine divergence times with the approximate likelihood calculation method and data from TimeTree [40]. The resulting tree suggests that Giraffidae are a sister taxon to the Cervidae, diverging ∼21.5 million years ago (Fig. 4); however, further studies using more deer species and other ruminants, such as pronghorn, as well as other methodologies to detect orthologous genes, will be needed to clarify the ruminant phylogeny.

## Conclusions

Herein, we report a *de novo* chromosome-scale genome assembly for Masai giraffe using a combination of sequencing and assembly methodologies aided by physical mapping of 153 BACs onto giraffe metaphase chromosomes. Gene and repeat annotation of the assembly identified a similar number of genes and transposable elements as found in other ruminant species. Following the example of the sable antelope [41] and the California condor [42], the new giraffe genome assembly will foster research into conservation of this charismatic species, serving as a foundation for characterizing the genetic diversity of wild and captive populations. Furthermore, the high-quality, chromosome-scale assembly described in this report contributes to the goals of the Genome 10K Project [43] and the Earth BioGenome Project [44].

## Availability of supporting data

The raw sequence data have been deposited in the Short Read Archive under accession numbers SRR7503131, SRR7503132, SRR7503129, SRR7503130, SRR7503127, SRR7503128, SRR7503125, SRR7503126, SRR7503158, SRR7503157, SRR7503156, and SRR7503155. The SOAPdenovo + Chicago assembly is also available in NCBI under accession number RAWU00000000. Annotations and chromosome reconstructions are available in the GigaScience database GigaDB [45].

## Note added in proof

The underlying giraffe SOAPdenovo assembly described in this article is the same as the one used by Chen et al. [46].

## Additional files

giraffe_SupplData_reviewerComments_DL.docx

SupplFig1.pdf

giz090_GIGA-D-19-00077_Original_Submission

giz090_GIGA-D-19-00077_Revision_1

giz090_GIGA-D-19-00077_Revision_2

giz090_Response_to_Reviewer_Comments_Original_Submission

giz090_Response_to_Reviewer_Comments_Revision_1

giz090_Reviewer_1_Report_Original_Submission Terje Raudsepp -- 3/31/2019 Reviewed

giz090_Reviewer_2_Report_Original_Submission Jane Loveland -- 4/1/2019 Reviewed

giz090_Reviewer_3_Report_Original_Submission Derek Bickhart -- 4/1/2019 Reviewed

giz090_Reviewer_4_Report_Original_Submission Morris Agaba -- 4/3/2019 Reviewed

giz090_Supplemental_Files

## Abbreviations

BCA: bacterial artificial chromosome; BUSCO: Benchmarking Universal Single-Copy Orthologs; FISH: fluorescence *in situ* hybridization; PCF: predicted chromosome fragment; RACA: Reference-Assisted Chromosome Assembly; SF: syntenic fragment; TE: transposable element.

## Competing interests

The authors declare that they have no competing interests.

## Funding

This work was supported in part by the US Department of Agriculture Cooperative State Research Education and Extension Service (Livestock Genome Sequencing Initiative Grants 538 AG2009-34480-19875 and 538 AG 58-1265-0-03 to H.A.L.), the Biotechnology and Biological Sciences Research Council (Grant BB/P020062/1 to D.M.L.), and Russian Foundation for Basic Research (RFBR) grants 17-00-00145 (D.M.L.) and 17-00-00146 (A.S.G.) as part of 17-00-00148 (K).

## Author contributions

M.F. generated the SOAPdenovo + RACA assembly, evaluated the assemblies, and cowrote the manuscript. I.D. performed PCR verifications and ran the adjusted parameters SOAPdenovo + RACA assembly. Q.L. and Y.Z. assembled the sequencing reads with SOAPdenovo and annotated the genome. J.D. performed paired-end read mapping and cowrote the manuscript. A.P., A.K., and A.S.G. performed FISH on giraffe chromosomes. L.G.C. and O.A.R. prepared cell cultures and extracted DNA. G.Z. supervised SOAPdenovo assembly and gene annotation. J.K. and J.M. assisted in RACA assemblies. D.M.L. and H.A.L. supervised the project and revised the manuscript.
