# Supplementary material for: An integrated chromosome-scale genome assembly of the Masai giraffe (Giraffa camelopardalis tippelskirchi)
Source: Gigascience. 2019 Jul 30;8(8):giz090. doi: 10.1093/gigascience/giz090 (PMC6669057; doi:10.1093/gigascience/giz090)
Supplement: giz090_GIGA-D-19-00077_Original_Submission [file giz090_giga-d-19-00077_original_submission.pdf]

GigaScience

An integrated chromosome-scale genome assembly of the Masai Giraffe (Giraffa camelopardalis tippelskirchi)

--Manuscript Draft--

**Manuscript Number:**	GIGA-D-19-00077
**Full Title:**	An integrated chromosome-scale genome assembly of the Masai Giraffe (Giraffa camelopardalis tippelskirchi)
**Article Type:**	Data Note
**Funding Information:**	Cooperative State Research, Education, and Extension Service (538 AG2009-34480-19875) — Prof Harris A. Lewin<br>Cooperative State Research, Education, and Extension Service (538 AG 58-1265-0-03) — Prof Harris A. Lewin<br>Biotechnology and Biological Sciences Research Council (GB) (BB/P020062/1) — Dr Dennis Larkin<br>Russian Foundation for Basic Research (17-00-00145) — Dr Dennis Larkin<br>Russian Foundation for Basic Research (17-00-00146) — Prof Alexander S. Graphodatsky

Funding source	Recipient
Cooperative State Research, Education, and Extension Service (538 AG2009-34480-19875)	Prof Harris A. Lewin
Cooperative State Research, Education, and Extension Service (538 AG 58-1265-0-03)	Prof Harris A. Lewin
Biotechnology and Biological Sciences Research Council (GB) (BB/P020062/1)	Dr Dennis Larkin
Russian Foundation for Basic Research (17-00-00145)	Dr Dennis Larkin
Russian Foundation for Basic Research (17-00-00146)	Prof Alexander S. Graphodatsky

Abstract:

Background

The Masai giraffe (Giraffa camelopardalis tippelskirchi) is the largest-bodied giraffe and the world's tallest terrestrial animal. With its extreme size and height, the giraffe's unique anatomical and physiological adaptations have long been of interest to diverse research fields. Giraffes are also critical to ecosystems of sub-Saharan Africa, with their long neck serving as a conduit to food sources not shared by other herbivores. Although the genome of a Masai giraffe has been sequenced, the assembly was highly fragmented and unsuitable for the analysis of chromosome evolution. Herein we report an improved giraffe genome assembly to facilitate evolutionary analysis of the giraffe and other ruminant genomes.

Findings

Using SOAPdenovo2 and 170 Gbp of Illumina paired-end and mate-pair reads we generated a 2.6 Gbp female Masai giraffe genome assembly, with a scaffold N50 of 3 Mbp. The incorporation of 114.6 Gbp of Chicago library sequencing data resulted in a HiRise SOAPdenovo + Chicago assembly with an N50 of 48 Mbp and containing 95% of expected genes according to BUSCO analysis. Using the Reference-Assisted Chromosome Assembly tool, we were able to order and orient scaffolds into 42 predicted chromosome fragments (PCFs). Using fluorescence in situ hybridization we hybridized 153 cattle BACs onto giraffe metaphase spreads to assess and place the PCFs on 14 giraffe autosome and 10 sex chromosome fragments. In this assembly, 21,621 protein-coding genes were identified and 1 Gbp of repetitive sequences annotated using both de novo and homology-based predictions.

Conclusions

Our results provide the first chromosome-scale genome assembly for the Masai giraffe. This assembly will be a valuable tool to elucidate the evolution and molecular basis of adaptive traits of the Giraffidae, and will provide a new genomic resource to assist conservation efforts.

**Corresponding Author:**	Denis Larkin<br><br>UNITED KINGDOM
**Corresponding Author Secondary Information:**	

Powered by Editorial Manager® and ProduXion Manager® from Aries Systems Corporation

Corresponding Author's Institution:	
Corresponding Author's Secondary Institution:	
First Author:	Marta Farré
First Author Secondary Information:	
Order of Authors:	Marta Farré
	Qiye Li
	Iulia Darolti
	Yang Zhou
	Joana Damas
	Anastasia A. Proskuryakova
	Anastasia I. Kulemzina
	Leona G. Chemnick
	Jaebum Kim
	Oliver A. Ryder
	Jian Ma
	Alexander S. Graphodatsky
	Guoije Zhang
	Dennis Larkin
	Harris A. Lewin
Order of Authors Secondary Information:	
Additional Information:	
Question	Response
Are you submitting this manuscript to a special series or article collection?	No
**Experimental design and statistics**<br><br>Full details of the experimental design and statistical methods used should be given in the Methods section, as detailed in our Minimum Standards Reporting Checklist. Information essential to interpreting the data presented should be made available in the figure legends.<br><br>Have you included all the information requested in your manuscript?	Yes
**Resources**<br><br>A description of all resources used,	Yes

including antibodies, cell lines, animals and software tools, with enough information to allow them to be uniquely identified, should be included in the Methods section. Authors are strongly encouraged to cite Research Resource Identifiers (RRIDs) for antibodies, model organisms and tools, where possible.<br><br>Have you included the information requested as detailed in our Minimum Standards Reporting Checklist?	
**Availability of data and materials**<br><br>All datasets and code on which the conclusions of the paper rely must be either included in your submission or deposited in publicly available repositories (where available and ethically appropriate), referencing such data using a unique identifier in the references and in the "Availability of Data and Materials" section of your manuscript.<br><br>Have you have met the above requirement as detailed in our Minimum Standards Reporting Checklist?	No
If not, please give reasons for any omissions below.<br><br>as follow-up to "**Availability of data and materials**<br><br>All datasets and code on which the conclusions of the paper rely must be either included in your submission or deposited in publicly available repositories (where available and ethically appropriate), referencing such data using a unique identifier in the references and in the "Availability of Data and Materials" section of your manuscript.	Genome annotations, phylogenetic tree and BUSCO summaries will be uploaded to GigaDB

Have you have met the above requirement as detailed in our Minimum Standards Reporting Checklist?<br><br>"	

Manuscript
Click here to download Manuscript giraffe_manuscript_03082019.docx

An integrated chromosome-scale genome assembly of the Masai Giraffe (*Giraffa camelopardalis tippelskirchi*)

Marta Farré[1,2], Qiye Li[3,4], Iulia Darolti[1,5], Yang Zhou[5,6], Joana Damas[1,7], Anastasia A. Proskuryakova [8,9], Anastasia I. Kulemzina[8], Leona G. Chemnick[10], Jaebum Kim[11], Oliver A. Ryder[10], Jian Ma[12], Alexander S. Graphodatsky[8,9], Guoije Zhang[3,4,6], Denis M. Larkin[1,13*] and Harris A. Lewin[7,14*]

1. Comparative Biomedical Sciences Department, Royal Veterinary College, University of London, London NW1 0TU, UK.
2. School of Biosciences, University of Kent, Canterbury CT2 7NJ, UK.
3. State Key Laboratory of Genetic Resources and Evolution, Kunming Institute of Zoology, Chinese Academy of Sciences, Kunming 650223, China.
4. China National Genebank, BGI-Shenzhen, Shenzhen 518083, China.
5. Department of Genetics, Evolution and Environment, University College London, London WC1E 6BT, UK.
6. Centre for Social Evolution, Department of Biology, Universitetsparken 15, University of Copenhagen, DK-2100 Copenhagen, Denmark.
7. The Genome Center, University of California, Davis, USA.
8. Institute of Molecular and Cellular Biology, SB RAS, Novosibirsk 630090, Russia.
9. Novosibirsk State University, Novosibirsk 630090, Russia.
10. San Diego Institute for Conservation Research, San Diego Zoo Global, Escondido, California, USA.
11. Department of Biomedical Science and Engineering, Konkuk University, Seoul 05029, South Korea.
12. Computational Biology Department, School of Computer Science, Carnegie Mellon University, USA.
13. The Federal Research Center Institute of Cytology and Genetics, The Siberian Branch of the Russian Academy of Sciences (ICG SB RAS), 630090, Novosibirsk, Russia.
14. Department of Evolution and Ecology, College of Biological Sciences, and the Department of Reproduction and Population Health, School of Veterinary Medicine, University of California, Davis, USA.

* Corresponding authors

Emails:
M.F.: mfarrebelmonte@gmail.com
Q.L.: liqiye@genomics.cn
I.D.: iulia.darolti.15@ucl.ac.uk
Y.Z.: zhouyang@genomics.cn
J.D.: joanadamas@gmail.com
A.A.P: andrena@mcb.nsc.ru
A.I.K.: zakal@mcb.nsc.ru
L.G.C.: lchemnick@sandiegozoo.org
J.K.: jbkim@konkuk.ac.kr
O.A.R.: oryder@sandiegozoo.org
J.M.: jianma@cs.cmu.edu
A.S.G.: graf@mcb.nsc.ru
G.Z.: zhanggj@genomics.cn
D.M.L.: dlarkin@rvc.ac.uk
H.A.L.: lewin@ucdavis.edu

Abstract

Background. The Masai giraffe (*Giraffa camelopardalis tippelskirchi*) is the largest-bodied giraffe and the world's tallest terrestrial animal. With its extreme size and height, the giraffe's unique anatomical and physiological adaptations have long been of interest to diverse research fields. Giraffes are also critical to ecosystems of sub-Saharan Africa, with their long neck serving as a conduit to food sources not shared by other herbivores. Although the genome of a Masai giraffe has been sequenced, the assembly was highly fragmented and unsuitable for the analysis of chromosome evolution. Herein we report an improved giraffe genome assembly to facilitate evolutionary analysis of the giraffe and other ruminant genomes. **Findings.** Using SOAPdenovo2 and 170 Gbp of Illumina paired-end and mate-pair reads we generated a 2.6 Gbp female Masai giraffe genome assembly, with a scaffold N50 of 3 Mbp. The incorporation of 114.6 Gbp of Chicago library sequencing data resulted in a HiRise SOAPdenovo + Chicago assembly with an N50 of 48 Mbp and containing 95% of expected genes according to BUSCO analysis. Using the Reference-Assisted Chromosome Assembly tool, we were able to order and orient scaffolds into 42 predicted chromosome fragments (PCFs). Using fluorescence in situ hybridization we hybridized 153 cattle BACs onto giraffe metaphase spreads to assess and place the PCFs on 14 giraffe autosome and 10 sex chromosome fragments. In this assembly, 21,621 protein-coding genes were identified and 1 Gbp of repetitive sequences annotated using both *de novo* and homology-based predictions. **Conclusions.** Our results provide the first chromosome-scale genome assembly for the Masai giraffe. This assembly will be a valuable tool to elucidate the evolution and molecular basis of adaptive traits of the Giraffidae, and will provide a new genomic resource to assist conservation efforts.

Keywords (3-10 words): giraffe, *Giraffa camelopardalis tippelskirchi*, assembly, annotation, ruminant

Background information

Giraffes (*Giraffa*) are a genus of even-toed ungulate mammals comprising four species [1]. They are members of the family Giraffidae, which also includes the okapi (*Okapia johnstoni*). The Masai giraffe (also known as Kilimanjaro giraffe; *Giraffa camelopardalis tippelskirchi*; Figure 1) is native to East Africa and distributed throughout Tanzania and Kenya [2]. Masai giraffes are not only the largest-bodied giraffes [3] but also the tallest terrestrial animals. Giraffes present several distinctive anatomical characteristics, such as their long neck and legs, horn-like ossicones and coat patterns,

which together with their unique cardiovascular and musculoskeletal adaptations have interested researchers in many fields [3-6].

The giraffe genome comprises 15 pairs of chromosomes (2n = 30) that are believed to have originated by multiple Robertsonian fusions from the pecoran ancestral karyotype (2n = 58) [7, 8]. In 2016, Agaba and colleagues generated the first genome sequence of a female Masai giraffe and compared it with the genome sequence of an okapi [9]. This study identified candidate genes and pathways involved in the giraffes' unique skeletal and cardiovascular adaptations [9]. The reported genome was very fragmented, which hinders its use for studies of overall genome architecture and evolution. Many missing and fragmented genes also limit the utility of the assembly for the study of the genetic basis for the giraffe's unique adaptations. Here we report a chromosome-scale assembly of a female Masai giraffe genome sequenced *de novo*. This assembly will facilitate studies of ruminant genome evolution and will be a powerful resource for further elucidation of the genetic basis for the giraffe's characteristic features. Furthermore, having another Masai giraffe genome sequence will assist conservation efforts of this species, whose population has declined by more than 52% in recent decades [2, 10].

Data description

Library construction, sequencing, and filtering

Genomic DNA was extracted from a fibroblast cell culture of a female Masai giraffe using the DNeasy Blood & Tissue Kit (QIAGEN, Valencia, CA, USA) according to the manufacturer's instructions. Isolated genomic DNA was then used to construct twelve sequencing libraries, four short-insert (170, 250, 500, and 800 bp) and eight long-insert size (2, 5, 10, and 20 Kbp), following Illumina (San Diego, CA, USA) standard protocols. Using a whole-genome shotgun sequencing strategy on the Illumina Hiseq 2000 platform, we generated 296.23 Gbp of raw sequencing data with 100 bp or 50 bp paired-end sequencing for the short-insert or long-insert size libraries, respectively (Supplementary Table 1). To improve read quality, low-quality bases from both ends of the reads were trimmed, duplicated reads and those with more than 5% of uncalled ("N") bases were removed. A total of 171.09 Gbp of filtered read data were used for genome assembly.

Two Chicago libraries were generated by Dovetail Genomics (Santa Cruz, CA) as previously described [11]. Briefly, high-molecular-weight DNA was assembled into chromatin *in vitro*, chemically cross-linked and digested by restriction enzymes. The resulting digestion overhangs were filled in with a biotinylated nucleotide, and the chromatin was incubated in a proximity-ligation reaction. The cross-links were then reversed, and the DNA purified from the chromatin. These libraries were sequenced

in one flow-cell lane using the Illumina HiSeq 4000 platform, resulting in the generation of ~385 million read pairs.

Evaluation of the genome size

The Masai giraffe genome size was estimated by k-mer analysis. A k-mer refers to an artificial sequence division of K nucleotides iteratively from sequencing reads. A raw sequence read with L bp contains (L-K+1) different k-mers of length K bp. K-mer frequencies can be calculated from the genome sequence reads and typically follow a Poisson distribution when plotted against the sequence depth gradient. The genome size, G, can then be calculated from the formula G=K_num/K_depth, where the K_num is the total number of k-mers, and K_depth denotes the depth of coverage of the k-mer with the highest frequency. For giraffe, at K=17, K_num was 75,710,429,964 and the K_depth was 30. Therefore, we estimated the genome size of *Giraffa camelopardalis tippelskirchi* to be 2.5 Gbp, comparable to the C-value of 2.7 and 2.9 reported for reticulated giraffe (*Giraffa camelopardalis reticulata*) [12]. All the filtered Illumina sequencing reads provided approximately 61.2x mean coverage of the genome, while the Chicago libraries' reads presented an estimated genome coverage of 88.4x.

Genome assembly

We applied SOAPdenovo (version 2.04) with default parameters to construct contigs and scaffolds as described previously [13]. All reads were aligned against each other to produce contigs which were further assembled in scaffolds using the paired-end information. The generated Masai giraffe genome assembly was 2.55 Gbp long, including 76.82 Mbp (3%) of unknown bases ("Ns"). The contig and scaffold N50 lengths were 21.78 Kbp and 3.00 Mbp, respectively (Table 1). To assess the assembly quality, approximately 90 Gbp (representing 35.6x genome coverage) high-quality short-insert size reads were aligned to the SOAPdenovo assembly using BWA (with parameters of -t 1 -I). A total of 98.9% reads could be mapped and covered 98.9% of the assembly, excluding gaps. Approximately 92% of these reads were properly paired, having an expected insert size associated with the libraries of origin.

To increase the contiguity of the Masai giraffe genome assembly we used the HiRise2.1 scaffolder [11] and sequence information from the Chicago libraries and SOAPdenovo assembly as inputs. The SOAPdenovo + Chicago assembly introduced a total of 56 breaks in 54 SOAPdenovo scaffolds, and formed 3,200 new scaffold joints, resulting in an increased scaffold N50 length of 57.20 Mbp (Table 1).

Evaluation of the SOAPdenovo genome assembly and PCR verification of putatively chimeric scaffolds

To identify putatively chimeric scaffolds, we utilized the Masai giraffe SOAPdenovo genome assembly to obtain predicted chromosome fragments (PCFs) using the Reference-Assisted Chromosome Assembly (RACA) software [14]. The RACA tool uses a combination of comparative information and sequencing data to order and orient scaffolds of target species and generated PCFs. Cattle (*Bos taurus*, bosTau6) and human (*Homo sapiens*, hg19) genomes were used as a reference and outgroup, respectively, and all the Illumina paired-end and mate-pair libraries were included in the RACA assembly. The read libraries were aligned to the SOAPdenovo scaffolds using Bowtie2 [15]. The cattle-giraffe and cattle-human pairwise alignments were performed using lastZ and UCSC Kent utilities [16] as previously described [14, 17]. The RACA software was used at a minimum resolution of 150 Kbp for syntenic fragment (SF) detection. Only SOAPdenovo scaffolds >10 Kbp were used as input for RACA, comprising 95% of the whole assembly length.

After an initial run of RACA with default parameters, we tested the structure of 32/41 (76%) SOAPdenovo scaffolds flagged as putatively chimeric. Chimerism was evaluated using PCR amplification across the RACA-defined split of SF joint boundaries (Supplementary Table 2). On the basis of results obtained using PCR and mapping of short- and long-insert size read libraries, we established 158x as the minimum physical coverage of reads mapped across the SF joint intervals that allowed separation of scaffolds that were likely to be chimeric from those that were likely to be authentic (Supplementary Table 2). This threshold was used to update the parameters of a second round of RACA (stage 2 RACA), which resulted in the generation of 47 PCFs, of which 13 were homologous to complete cattle chromosomes. The stage 2 RACA assembly had an N50 length of 85.22 Mbp. This assembly comprised 1,283 SOAPdenovo scaffolds, representing 93% of the original SOAPdenovo assembly, of which 33 were split by RACA, and two were manually split as they had been shown to be chimeric by PCR (Table 1). These results indicate the power of comparative information for improving contiguity and for identification of problematic regions in *de novo* assemblies.

Evaluation of the HiRise SOAPdenovo + Chicago assembly

More than 94% of the joints introduced in the SOAPdenovo + Chicago assembly were concordant with the RACA assembly, 4% were inconsistent between the two assemblies, and 1% represented extra adjacencies with intervening scaffolds located at the ends of PCFs. Among the 54 SOAPdenovo scaffolds broken in the SOAPdenovo + Chicago assembly, 26 were also broken in the RACA assembly. Among the remaining 28 scaffolds, five were not included within PCFs because they were under the 150 Kbp SF resolution set in the RACA tool; 16 were broken in the Chicago assembly, with one of the fragments below SF resolution, and seven scaffolds were broken in the SOAPdenovo + Chicago

assembly and intact in the RACA assembly (SOAPdenovo scaffolds 82, 813, 816, 849, 906, 940, and 995). Additionally, among the 16 SOAPdenovo scaffolds PCR-verified to be chimeric, 13 were broken in the SOAPdenovo + Chicago assembly. The remaining three chimeric joints, within SOAPdenovo scaffolds 181, 267, and 696 were manually split in the SOAPdenovo + Chicago assembly (scaffolds Sc_7219;HRSCAF=8761 and Sc_732785;HRSCAF=735706). The final SOAPdenovo + Chicago genome assembly comprises 2.6 Gbp and has an N50 length of 47.97 Mbp (Table 1).

Five chromosomal fusions as compared to cattle chromosomes were identified in the SOAPdenovo + Chicago assembly. Two of those fusions, cattle chromosomes (BTA1/BTA28 and BTA26/BTA28), were previously detected using cytogenetic approaches, and both locate on *Giraffa camelopardalis* chromosome 2 [7, 8]. Finally, we ran RACA using the SOAPdenovo + Chicago scaffolds and cattle (bosTau6) and human (hg19) genomes as reference and outgroup, respectively. RACA produced 42 PCFs, 20 of them representing complete cattle chromosomes (Table 1), a substantial improvement over the SOAPdenovo + RACA assembly.

Evaluation of SOAPdenovo + Chicago + RACA assembly and placement into chromosomes using FISH

In order to assess and map the SOAPdenovo + Chicago + RACA PCFs onto giraffe chromosomes, we performed fluorescence *in situ* hybridization (FISH) of cattle bacterial artificial chromosomes (BACs) from CHORI-240 library (http://www.chori.org/bacpac) with giraffe metaphase spreads (Figure 2) following previous publications [18]. Briefly, giraffe fibroblast cells were incubated at 37°C and 5% CO_2 in Alpha MEM (Gibco) supplemented with 15% Fetal Bovine Serum (Gibco), 5% AmnioMAX-II (Gibco) and antibiotics (ampicillin 100 µg/ml, penicillin 100 µg/ml, amphotericin B 2.5 µg/ml). Metaphases were obtained by adding colcemid (0.02 mg/ml) and EtBr (1.5 mg/ml) to actively dividing cultures. Hypotonic treatment was performed with KCl (3 mM) and sodium citrate (0.7 mM) for 20 min at 37°C and followed by fixation with 3:1 methanol-glacial acetic acid fixative. BAC DNA was isolated using a plasmid DNA isolation kit (Biosilica, Novosibirsk, Russia) and amplified using whole genome amplification (GenomePlex Whole Genome Amplification Kit, Sigma). Labeling of BAC DNA was performed using the GenomePlex WGA Reamplification Kit (Sigma) by incorporating biotin- 16-dUTP (Roche) or digoxigenin-dUTP (Roche). Two color FISH experiments on G-banded metaphase chromosomes were performed as described previously [18].

Cattle (bosTau6) assembly BAC clone coordinates were downloaded from NCBI CloneDB [19] and converted to coordinates in the giraffe SOAPdenovo + Chicago + RACA PCFs using the UCSC Genome Browser LiftOver tool [20]. A total of 153 BACs were successfully mapped to the giraffe assembly and were retained for the following analysis. To evaluate the 146 scaffold joints introduced by RACA, a reliability score was further calculated considering four components: (i) the relative

positions of the BACs in giraffe metaphase spreads compared to the PCFs (Figure 2), (ii) the joint was supported by sequence reads from Chicago libraries, (iii) physical coverage of Illumina pair-end reads, and (iv) comparative syntenic information. Different weights were given to each component of the score, ranging from 10% for the comparative syntenic information to 40% for the physical map using BAC data (Supplementary Table 3). Only those joints with a reliability score >30% were considered as authentic, indicating that at least FISH or Chicago support was present. More than 89% (N=130) of the adjacencies had FISH and/or Chicago support, while six (4%) adjacencies had syntenic support only (Supplementary Figure 1).

Completeness evaluation of genome assemblies using BUSCO

We evaluated genome completeness using the Benchmarking Universal Single-Copy Orthologs (BUSCO; version 3.0; [21]) software. Approximately 95% of the core mammalian gene set was complete in the SOAPdenovo and SOAPdenovo + Chicago assemblies; SOAPdenovo + RACA included 93% of the mammalian gene set, while the final chromosome-level assembly contained 95% complete BUSCO genes, similar to other reference-level ruminant assemblies (94% for cattle ARS-UCD1.2 and goat ARS1). In comparison, the Masai giraffe genome assembly reported by Agaba and colleagues [9] included 87% of BUSCO genes (Figure 3). These results show that the genome assemblies we generated are of high completeness and accuracy, and a significant improvement over the genome assembly currently available for Masai giraffe. Furthermore, the high quality, chromosome scale assembly described in this report contributes to the goals of the Genome 10K Project [22] and the Earth BioGenome Project [23].

Genome annotation

To annotate transposable elements (TEs) in the Masai giraffe genome, we started by predicting TEs by homology to RepBase sequences using RepeatProteinMask and RepeatMasker [24] with default parameters. Results from both types of software were combined to produce a non-redundant final set of TEs. Approximately 40% of the Masai giraffe's genome is comprised of TEs, with LINEs being the most frequent group (24%, Supplementary Table 4).

The remainder of the SOAPdenovo genome assembly was annotated using both homology-based and *de novo* methods. For the homology-based prediction, human, mouse, cow, and horse proteins were downloaded from Ensembl (release 64) and mapped onto the genome using tblastn. The homologous genome sequences were aligned against the matching proteins using Genewise [25] to define gene models. For *de novo* prediction, Augustus [26], Genscan [27], and SNAP [28] were applied to predict coding genes as described in [29]. Finally, homology-based and *de novo* derived gene sets

were merged to form a comprehensive and non-redundant reference gene set using GLEAN [30]. We obtained a reference gene set that contained 21,621 genes (Supplementary Table 5).

To assign functions to the newly annotated genes in the Masai giraffe genome we aligned them to SwissProt database using blastp with an (E)- value cutoff of 1 e^{-5}. A total of 18,910 genes (87.46% of the total annotated genes) had a Swissprot match. Publicly available databases (including Pfam, PRINTS, PROSITE, ProDom, and SMART) were used to annotate motifs and domains in the gene sequences using InterPro, producing a total of 16,137 genes annotated with domain information (74.64%). By searching the KEGG database using a best hit for each gene, 9,087 genes were mapped to a known pathway (42.03% of the genes). Finally, we assigned a gene ontology term to 12,263 genes, representing 56.72% of the full gene set. Overall, 18,955 genes (87.67%) had at least one functional annotation (Supplementary Table 6).

Genome evolution

The position of the Giraffidae family in the Ruminantia has been highly debated, with some studies using mitochondrial DNA or SNPchip data suggesting that Giraffidae are an outgroup to Bovidae and Cervidae [31, 32], while palaeontological and biochemical evidence [33, 34] suggested that Giraffidae and Cervidae are sister taxa. To shed light on the giraffe phylogeny, we first used the TreeFam methodology [35] to define gene families in eight mammalian genomes (cattle, sheep, gemsbok, yak, giraffe, Pere David's deer, horse and human) using newly defined or available gene annotations. We applied the same pipeline and parameters as described by Kim and co-workers [36]. A total of 16,148 gene families, of which 1,327 are single-copy orthologous families, were obtained. Concatenated protein sequence alignments of single-copy orthologous families were used as input for building the tree, with the JTT+gamma model, using PhyMLv3.3 [37]. Branch reliability was assessed by 1,000 bootstrap replicates. Finally, PAML mcmctree [38] was used to determine divergence times with the approximate likelihood calculation method and data from TimeTree [39]. The resulting tree suggests that Giraffidae are a sister taxon to the Cervidae, diverging ~21.5 million year ago (Figure 4); however, further studies using more deer species and other ruminants, such as pronghorn, as well as other methodologies to detect orthologous genes, will be needed to clarify the ruminant phylogeny.

List of abbreviations

BUSCO: Benchmarking Universal Single-Copy Orthologs; PCF: Predicted Chromosome Fragment; RACA: Reference-Assisted Chromosome Assembly; TE: Transposable Element.

Availability of supporting data

The raw sequence data have been deposited in the Short Read Archive (SRA) under accession numbers SRR7503131, SRR7503132, SRR7503129, SRR7503130, SRR7503127, SRR7503128, SRR7503125, SRR7503126, SRR7503158, SRR7503157, SRR7503156, SRR7503155. The SOAPdenovo + Chicago assembly is also available in NCBI under accession number RAWU00000000. Annotations and chromosome reconstructions are available in the *GigaScience* database.

Competing interests

The authors declare that they have no competing interests.

Funding

This work was supported in part by the US Department of Agriculture Cooperative State Research Education and Extension Service (Livestock Genome Sequencing Initiative Grants 538 AG2009-34480-19875 and 538 AG 58-1265-0-03 to H.A.L), the Biotechnology and Biological Sciences Research Council (Grant BB/P020062/1 to D.M.L.), and Russian Foundation for Basic Research (RFBR) grants 17-00-00145 (D.M.L.) and 17-00-00146 (A.S.G.) as part of 17-00-00148 (K).

Author contributions

M.F. generated the SOAPdenovo + RACA assembly, evaluated the assemblies, and co-wrote the manuscript. I.D. performed PCR verifications and ran the adjusted parameters SOAPdenovo + RACA assembly. Q.L. and Y.Z. assembled the sequencing reads with SOAPdenovo and annotated the genome. J.D. performed paired-end read mapping and co-wrote the manuscript. A.P., A.K. and A.G. performed FISH on giraffe chromosomes. L.G.C. and O.A.R. prepared cell cultures and extracted DNA. G.Z. supervised SOAPdenovo assembly and gene annotation. J.K. and J.M. assisted in RACA assemblies. D.M.L. and H.A.L. supervised the project and revised the manuscript.

Acknowledgments

We thank Prof John Hutchinson from the Royal Veterinary College (UK) and Prof Terence J. Robinson from Stellenbosch University (South Africa) for access to giraffe tissue materials.

References

1. Fennessy J, Bidon T, Reuss F, Kumar V, Elkan P, Nilsson MA, et al. Multi-locus Analyses Reveal Four Giraffe Species Instead of One. Current biology : CB. 2016;26 18:2543-9. doi:10.1016/j.cub.2016.07.036.

2. Muller Z, Bercovitch F, Brand R, Brown D, Brown M, Bolger D, et al. Giraffa camelopardalis. The IUCN Red List of Threatened Species 2016. 2016.
3. Dagg AI. Giraffe: Biology, Behaviour and Conservation. Cambridge University Press; 2014.
4. Solounias N. The remarkable anatomy of the giraffe's neck. Journal of Zoology. 1999;247 2:257-68. doi:undefined.
5. Estes R. The Behavior Guide to African Mammals: Including Hoofed Mammals, Carnivores, Primates. University of California Press; 1991.
6. Nowak RM. Walker's Mammals of the World. Johns Hopkins University Press; 1999.
7. Huang L, Nesterenko A, Nie W, Wang J, Su W, Graphodatsky AS, et al. Karyotype evolution of giraffes (Giraffa camelopardalis) revealed by cross-species chromosome painting with Chinese muntjac (Muntiacus reevesi) and human (Homo sapiens) paints. Cytogenetic and genome research. 2008;122 2:132-8. doi:10.1159/000163090.
8. Cernohorska H, Kubickova S, Kopecna O, Kulemzina AI, Perelman PL, Elder FF, et al. Molecular cytogenetic insights to the phylogenetic affinities of the giraffe (Giraffa camelopardalis) and pronghorn (Antilocapra americana). Chromosome research : an international journal on the molecular, supramolecular and evolutionary aspects of chromosome biology. 2013;21 5:447-60. doi:10.1007/s10577-013-9361-0.
9. Agaba M, Ishengoma E, Miller WC, McGrath BC, Hudson CN, Bedoya Reina OC, et al. Giraffe genome sequence reveals clues to its unique morphology and physiology. Nature communications. 2016;7:11519. doi:10.1038/ncomms11519.
10. Bolger DT, Ogutu JO, Strauss M, Lee DE, Fennessy J and Brown D. Masai giraffe (Giraffa camelopardalis tippelskirchi) conservation status report. IUCN/SSC Giraffe and Okapi Specialist Group. 2015.
11. Putnam NH, O'Connell BL, Stites JC, Rice BJ, Blanchette M, Calef R, et al. Chromosome-scale shotgun assembly using an in vitro method for long-range linkage. Genome Res. 2016;26 3:342-50. doi:10.1101/gr.193474.115.
12. 2017. http://www.genomesize.com.
13. Luo R, Liu B, Xie Y, Li Z, Huang W, Yuan J, et al. SOAPdenovo2: an empirically improved memory-efficient short-read de novo assembler. Gigascience. 2012;1 1:18. doi:10.1186/2047-217x-1-18.
14. Kim J, Larkin DM, Cai Q, Cai Q, Zhang Y, Ge R-L, et al. Reference-assisted chromosome assembly. Proc Natl Acad Sci U S A. 2013;110 5:1785-90. doi:10.1073/pnas.1220349110.
15. Langmead B and Salzberg SL. Fast gapped-read alignment with Bowtie 2. Nat Methods. 2012;9 4:357-9. doi:10.1038/nmeth.1923.
16. Kent WJ, Sugnet CW, Furey TS, Roskin KM, Pringle TH, Zahler AM, et al. The human genome browser at UCSC. Genome Res. 2002;12 6:996-1006. doi:10.1101/gr.229102. Article published online before print in May 2002.
17. Damas J, O'Connor R, Farre M, Lenis VP, Martell HJ, Mandawala A, et al. Upgrading short read animal genome assemblies to chromosome level using comparative genomics and a universal probe set. Genome Res. 2016; doi:10.1101/gr.213660.116.
18. Proskuryakova AA, Kulemzina AI, Perelman PL, Makunin AI, Larkin DM, Farré M, et al. X Chromosome Evolution in Cetartiodactyla. Genes. 2017;8 9:216. doi:10.3390/genes8090216.
19. Schneider VA, Chen HC, Clausen C, Meric PA, Zhou Z, Bouk N, et al. Clone DB: an integrated NCBI resource for clone-associated data. Nucleic acids research. 2013;41 Database issue:D1070-8. doi:10.1093/nar/gks1164.

20. Karolchik D, Baertsch R, Diekhans M, Furey TS, Hinrichs A, Lu YT, et al. The UCSC Genome Browser Database. Nucleic acids research. 2003;31 doi:10.1093/nar/gkg129.
21. Simao FA, Waterhouse RM, Ioannidis P, Kriventseva EV and Zdobnov EM. BUSCO: assessing genome assembly and annotation completeness with single-copy orthologs. Bioinformatics. 2015;31 19:3210-2. doi:10.1093/bioinformatics/btv351.
22. Koepfli K-P, Benedict Paten, Scientists tGKCo and O'Brien SJ. The Genome 10K Project: A Way Forward. Annual Review of Animal Biosciences. 2015;3 1:57-111. doi:doi:10.1146/annurev-animal-090414-014900.
23. Lewin HA, Robinson GE, Kress WJ, Baker WJ, Coddington J, Crandall KA, et al. Earth BioGenome Project: Sequencing life for the future of life. Proceedings of the National Academy of Sciences. 2018; doi:10.1073/pnas.1720115115.
24. Tarailo-Graovac M and Chen N. Using RepeatMasker to identify repetitive elements in genomic sequences. Curr Protoc Bioinformatics. 2009;Chapter 4:Unit 4.10. doi:10.1002/0471250953.bi0410s25.
25. Birney E, Clamp M and Durbin R. GeneWise and Genomewise. Genome Res. 2004;14 5:988-95. doi:10.1101/gr.1865504.
26. Stanke M, Keller O, Gunduz I, Hayes A, Waack S and Morgenstern B. AUGUSTUS: ab initio prediction of alternative transcripts. Nucleic Acids Res. 2006;34 Web Server issue:W435-9. doi:10.1093/nar/gkl200.
27. Burge C and Karlin S. Prediction of complete gene structures in human genomic DNA. J Mol Biol. 1997;268 1:78-94. doi:10.1006/jmbi.1997.0951.
28. Korf I. Gene finding in novel genomes. BMC Bioinformatics. 2004;5:59. doi:10.1186/1471-2105-5-59.
29. Zhang C, Chen L, Zhou Y, Wang K, Chemnick LG, Ryder OA, et al. Draft genome of the milu (Elaphurus davidianus). GigaScience. 2018;7 2:gix130-gix. doi:10.1093/gigascience/gix130.
30. Elsik CG, Mackey AJ, Reese JT, Milshina NV, Roos DS and Weinstock GM. Creating a honey bee consensus gene set. Genome Biol. 2007;8 1:R13. doi:10.1186/gb-2007-8-1-r13.
31. Hassanin A, Delsuc F, Ropiquet A, Hammer C, Jansen van Vuuren B, Matthee C, et al. Pattern and timing of diversification of Cetartiodactyla (Mammalia, Laurasiatheria), as revealed by a comprehensive analysis of mitochondrial genomes. Comptes rendus biologies. 2012;335 1:32-50. doi:10.1016/j.crvi.2011.11.002.
32. Decker JE, Pires JC, Conant GC, McKay SD, Heaton MP, Chen K, et al. Resolving the evolution of extant and extinct ruminants with high-throughput phylogenomics. Proceedings of the National Academy of Sciences. 2009;106 44:18644-9. doi:10.1073/pnas.0904691106.
33. Mitchell G and Skinner JD. On the origin, evolution and phylogeny of giraffes Giraffa camelopardalis. Transactions of the Royal Society of South Africa. 2003;58 1:51-73. doi:10.1080/00359190309519935.
34. Irwin DM, Kocher TD and Wilson AC. Evolution of the cytochrome b gene of mammals. Journal of molecular evolution. 1991;32 2:128-44.
35. Li H, Coghlan A, Ruan J, Coin LJ, Heriche JK, Osmotherly L, et al. TreeFam: a curated database of phylogenetic trees of animal gene families. Nucleic acids research. 2006;34 Database issue:D572-80. doi:10.1093/nar/gkj118.
36. Kim EB, Fang X, Fushan AA, Huang Z, Lobanov AV, Han L, et al. Genome sequencing reveals insights into physiology and longevity of the naked mole rat. Nature. 2011;479 7372:223-7. doi:10.1038/nature10533.

37. Guindon S, Dufayard JF, Lefort V, Anisimova M, Hordijk W and Gascuel O. New algorithms and methods to estimate maximum-likelihood phylogenies: assessing the performance of PhyML 3.0. Syst Biol. 2010;59 3:307-21. doi:10.1093/sysbio/syq010.
38. Yang Z. PAML 4: phylogenetic analysis by maximum likelihood. Mol Biol Evol. 2007;24 8:1586-91. doi:10.1093/molbev/msm088.
39. Hedges SB, Dudley J and Kumar S. TimeTree: a public knowledge-base of divergence times among organisms. Bioinformatics (Oxford, England). 2006;22 23:2971-2. doi:10.1093/bioinformatics/btl505.

Table 1. Assembly statistics of the *Giraffa camelopardalis tippelskirchi* genome.

	SOAPdenovo	SOAPdenovo + Chicago	SOAPdenovo + RACA	SOAPdenovo + Chicago + RACA	FINAL assembly
**Total length (Mbp)**	2,551.62	2,554.82	2,391.72	2,425.09	2,437.09
**N50 (Mbp)**	3.00	57.20	85.22	88.36	177.94
**No. scaffolds/PCFs**	739,028	735,884	47	42	24
**No. input scaffolds/PCFs broken**	--	54	33	16	0

Figure 1. An adult female Masai giraffe (*Giraffa camelopardalis tippelskirchi*) in the Masaai Mara national park, Kenya. Picture taken by Bjørn Christian Tørrissen, licence CC BY-SA 3.0.

Figure 2. Syntenic relationships between giraffe and cattle genomes. (A) Circos plot showing syntenic relationships between cattle autosomes (labelled as BTA) and giraffe chromosomes. Chromosomes are colored based on cattle homologies. Ribbons inside the plot show syntenic relationships, while lines inside each ribbon indicate inversions. (B) Placement of cattle BACs onto the giraffe karyotype. The first column of numbers on the right of each pair of giraffe chromosomes correspond to cattle (BTA) chromosomes, while the second column locates the cattle BAC IDs hybridized to giraffe chromosomes. (C) Giraffe chromosome 14 showing homologous synteny blocks (HSBs) between giraffe and cattle. SOAPdenovo and SOAPdenovo + Chicago scaffolds are also displayed. Blue blocks indicate positive (+) orientation of tracks compared with the giraffe chromosome while red blocks, negative (−) orientation. Numbers inside each block represent cattle chromosomes or giraffe scaffold IDs. BTA: *Bos taurus*, cattle. Images of all giraffe chromosomes could be found in Supplementary Fig. 1.

Figure 3. BUSCO assessment results. The BUSCO dataset of the mammalia_odb9 including 4,104 BUSCOs was used to assess the completeness of the four giraffe genome assemblies.

Figure 4. Phylogenetic relationships of the giraffe. Phylogenetic tree constructed with orthologous genes. Divergence times were extracted from the TimeTree database for calibration. Blue bars indicate the estimated diverge times in millions of years, and red circle indicates the calibration time.

Figure 1

Click here to download Figure Figure1.JPG

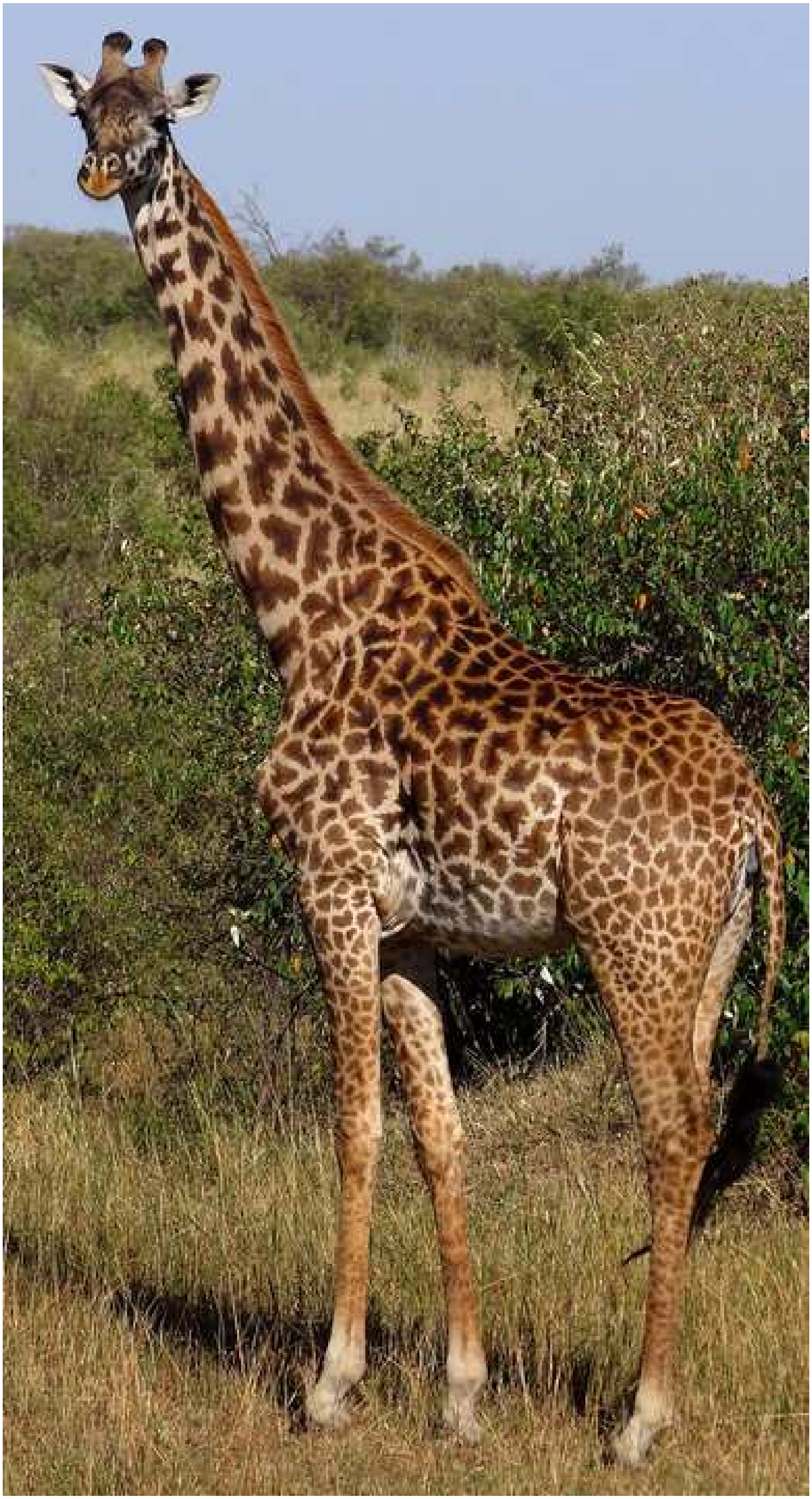

Figure 2

Click here to download Figure Figure2.pdf

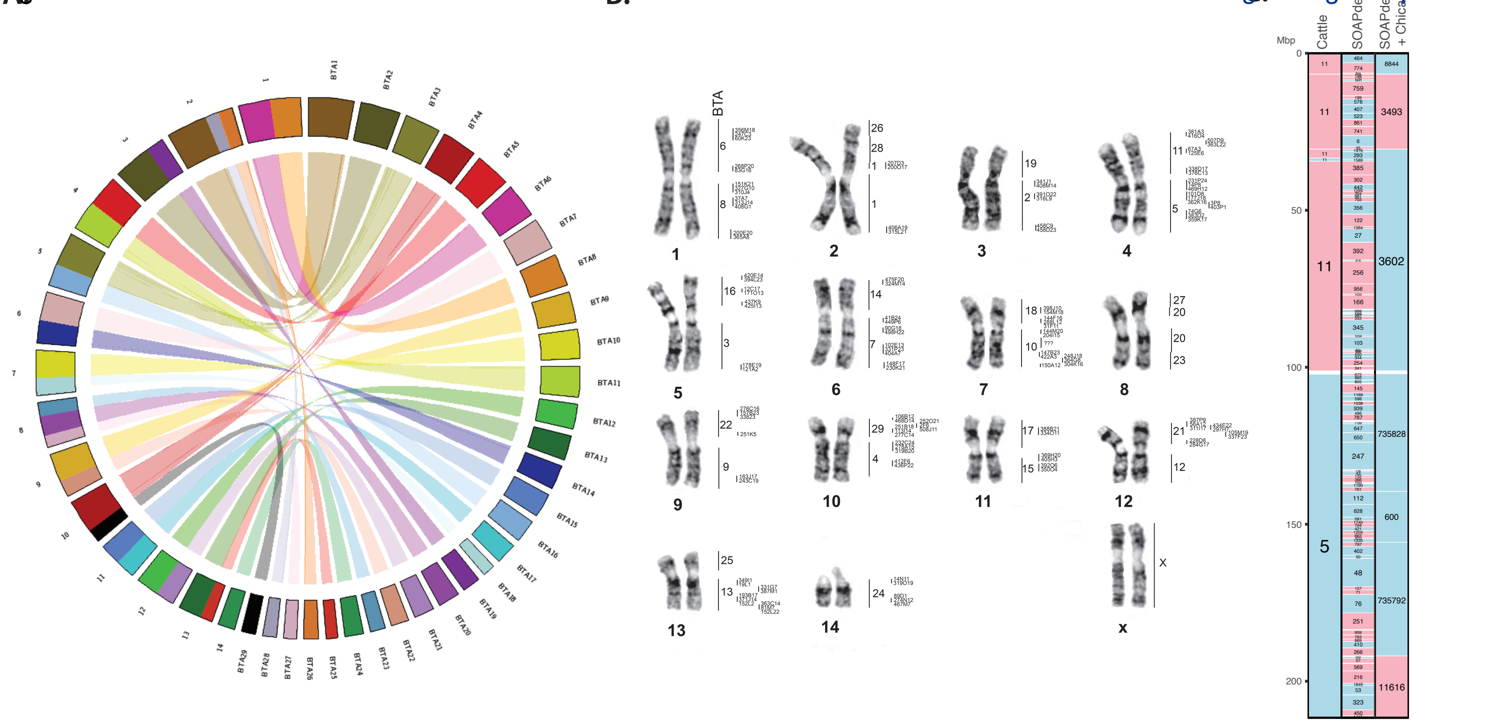

Figure 3

Click here to download Figure Figure3.pdf

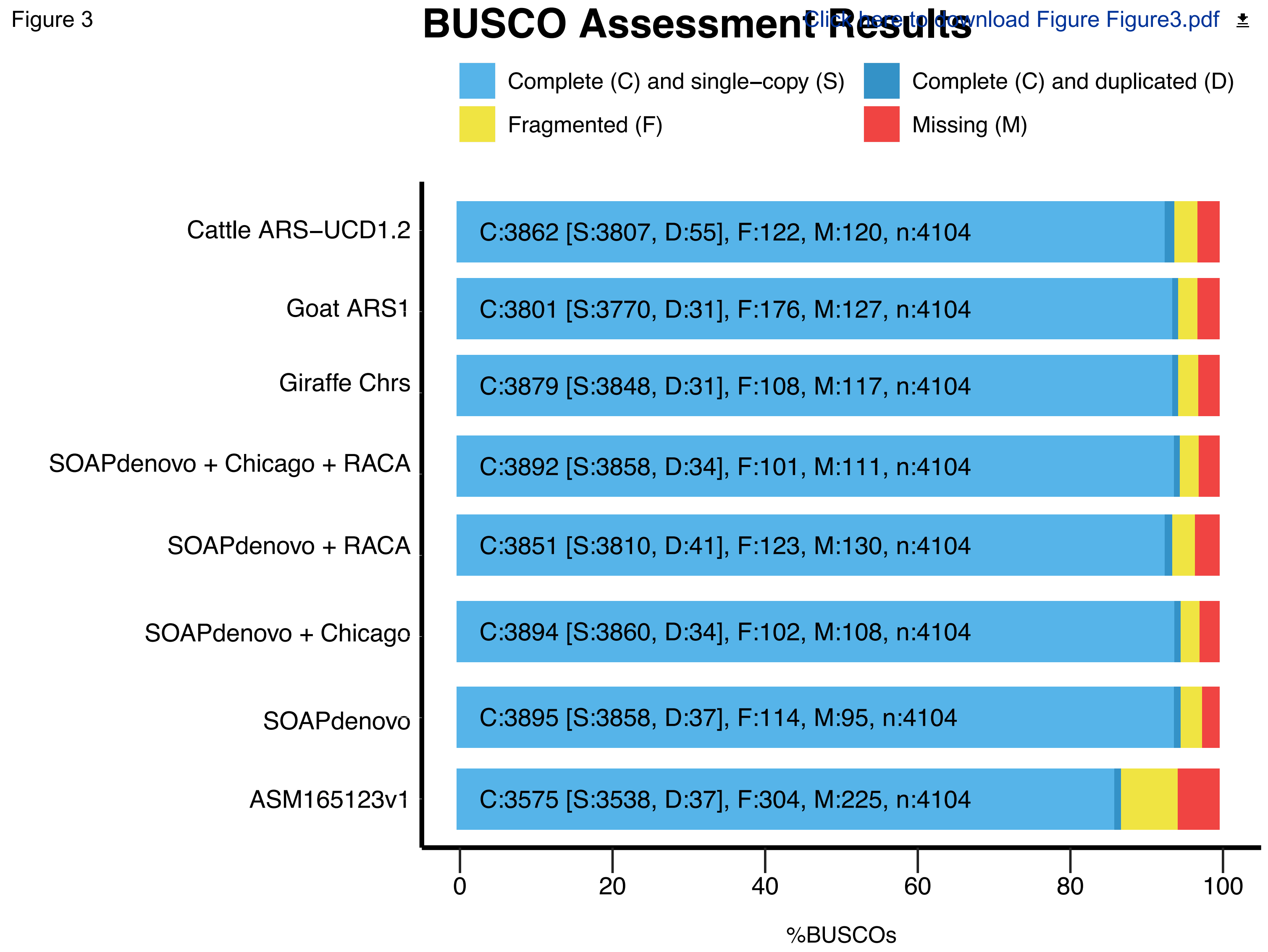

Figure 4

Click here to download Figure Figure4.pdf

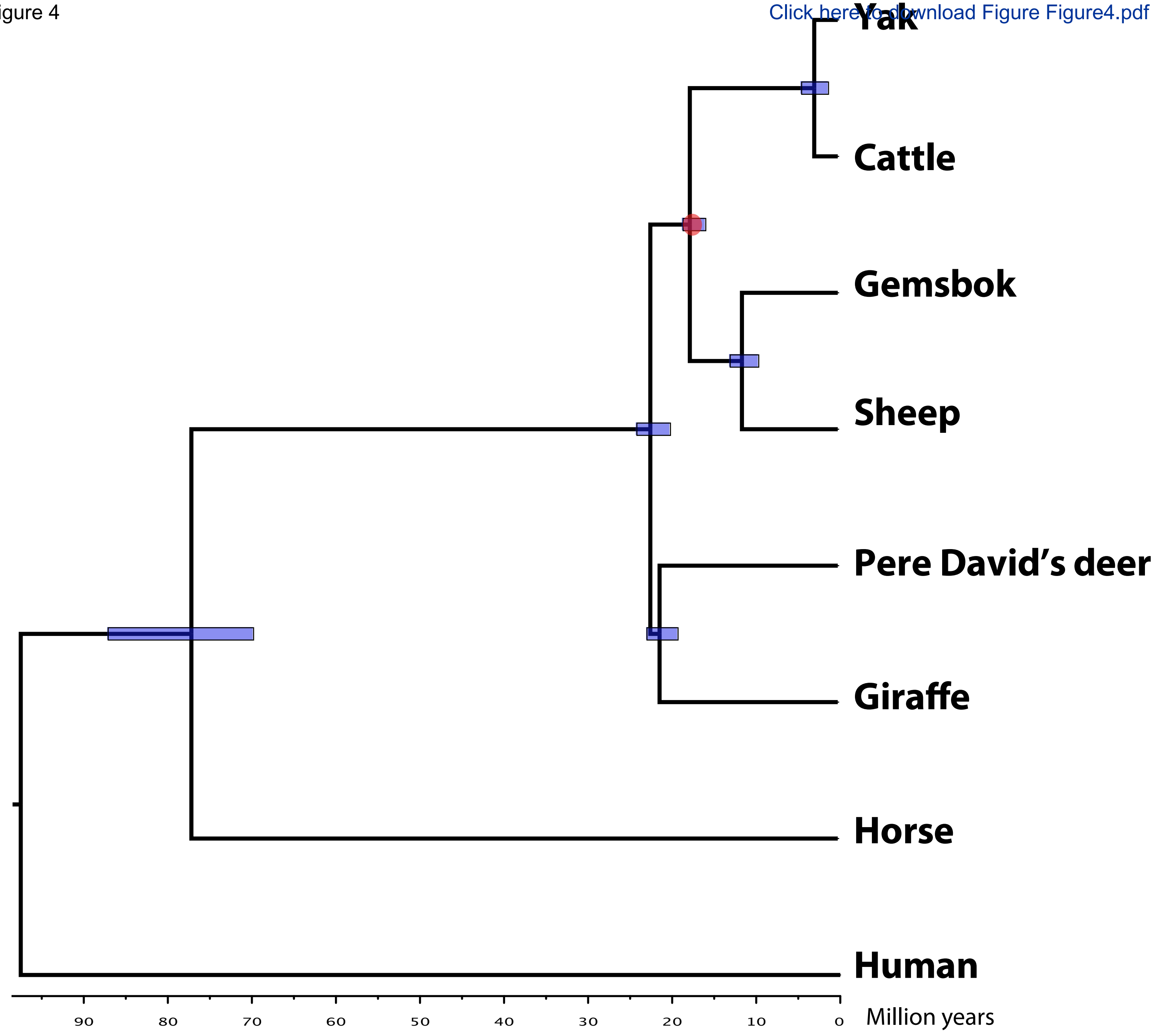

Click here to access/download
Supplementary Material
giraffe_SupplData.docx

Supplementary Figure 1

Click here to access/download
Supplementary Material
SupplFig1.pdf

Dear Editor,

We are submitting the manuscript entitled **"An integrated chromosome-scale genome assembly of the Masai Giraffe (*Giraffa camelopardalis tippelskirchi*)"** by Farré and coworkers for your consideration to be published in *GigaScience*.

The Masai giraffe (*Giraffa camelopardalis tippelskirchi*) is the largest-bodied giraffe and the world's tallest terrestrial animal. With its extreme size and height, the giraffe's unique anatomical and physiological adaptations have long been of interest to diverse research fields. Giraffes are also critical to ecosystems of sub-Saharan Africa, with their long neck serving as a conduit to food sources not shared by other herbivores. Although the genome of a Masai giraffe has been sequenced, the assembly was highly fragmented and unsuitable for the analysis of chromosome evolution. Here we report an improved giraffe genome assembly to facilitate evolutionary analysis of the giraffe and other ruminant genomes.

To achieve a chromosome assembly level, we used a hybrid approach, generating first NGS scaffold-level assembly. Then, sequenced Dovetail Chicago libraries were used to increase continuity of the assembly. And the Reference Assisted Chromosome Assembly tool was used to assess and lengthen the final assembly. And finally, fluorescent *in situ* hybridization (FISH) of 153 BACs was used to anchor and orient the chromosomal fragments into giraffe chromosomes. Using this hybrid approach, we were able to assemble the giraffe genome into 14 autosomes and 10 large sex chromosome fragments, containing more than 95% of the mammalian QC gene set.

We are committed to open science, and as such, all data produced during this project (pair-end read libraries, gene annotations and evaluation of the assemblies) are freely available from SRA and NCBI, with the accession numbers provided in the manuscript.

We believe that our paper will be of a broad interest to all readers interested in genomics and conservation biology. As such, we would like to suggest two potential reviewers for this manuscript:

a) Dr Klaus-Peter Koepfli, from the Smithsonian Conservation Biology Institute. Email: klauspeter.koepfli527@gmail.com.
b) Prof Terje Raudsepp, from Veterinary Medicine and Biomedical Sciences, Texas A&M University. Email: traudsepp@cvm.tamu.edu

All authors declare no competing interests. Thank you in advance for considering this manuscript.

Sincerely yours,
Harris Lewin
Denis Larkin
Marta Farré
