# Supplementary material for: An integrated chromosome-scale genome assembly of the Masai giraffe (Giraffa camelopardalis tippelskirchi)
Source: Gigascience. 2019 Jul 30;8(8):giz090. doi: 10.1093/gigascience/giz090 (PMC6669057; doi:10.1093/gigascience/giz090)
Supplement: giz090_Supplemental_Files [file giz090_supplemental_files.zip › SupplFig1.pdf]

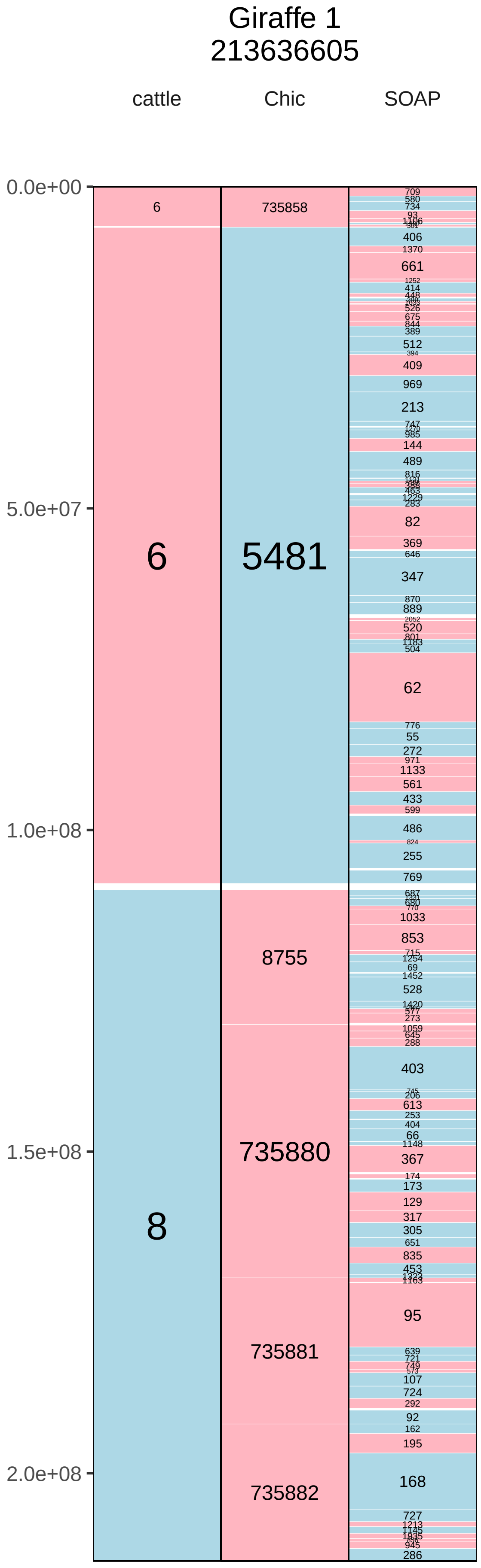

Giraffe 1
213636605
cattle
Chic
SOAP
0.0e+00
5.0e+07
1.0e+08
1.5e+08
2.0e+08
6
8
735858
5481
8755
735880
735881
735882
406
661
512
409
969
213
144
489
82
369
347
889
520
62
55
272
1133
561
433
486
255
769
1033
853
528
403
613
367
173
129
317
305
835
95
107
724
92
195
168
727
286

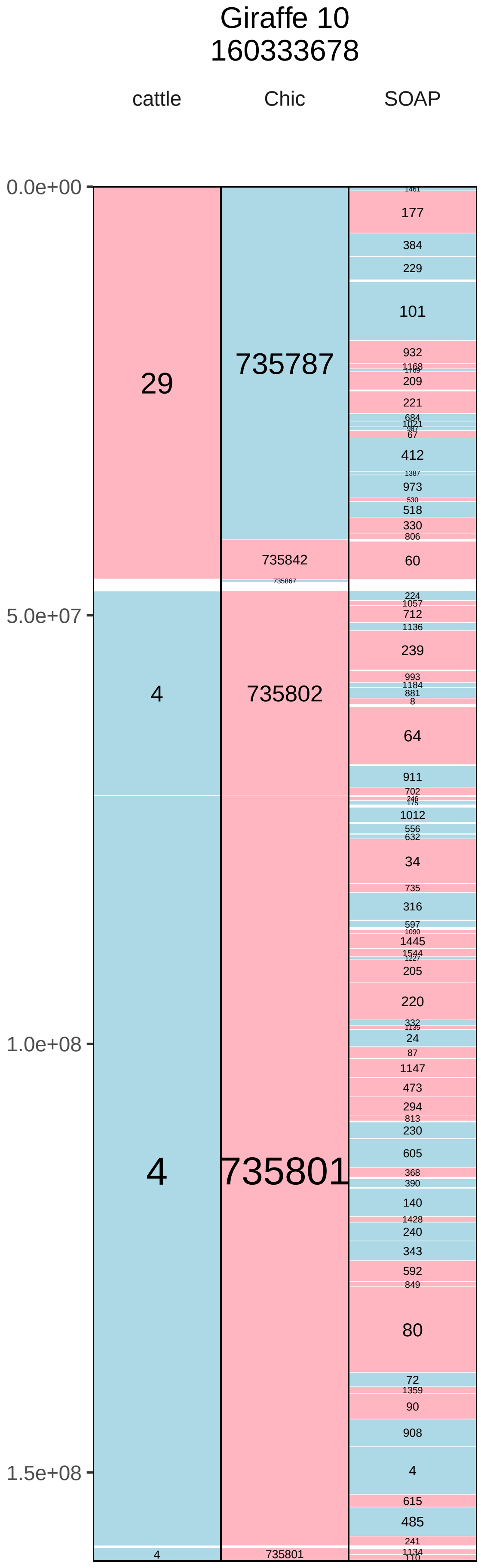

Giraffe 10
160333678
cattle
Chic
SOAP
0.0e+00
5.0e+07
1.0e+08
1.5e+08
29
4
4
4
735787
735842
735867
735802
735801
735801
177
384
229
101
932
1168
209
221
684
1021
67
412
1387
973
530
518
330
806
60
224
1057
712
1136
239
993
1184
881
8
64
911
702
246
175
1012
556
632
34
735
316
597
1090
1445
1544
1227
205
220
332
1135
24
87
1147
473
294
813
230
605
368
390
140
1428
240
343
592
849
80
72
1359
90
908
4
615
485
241
1134
110

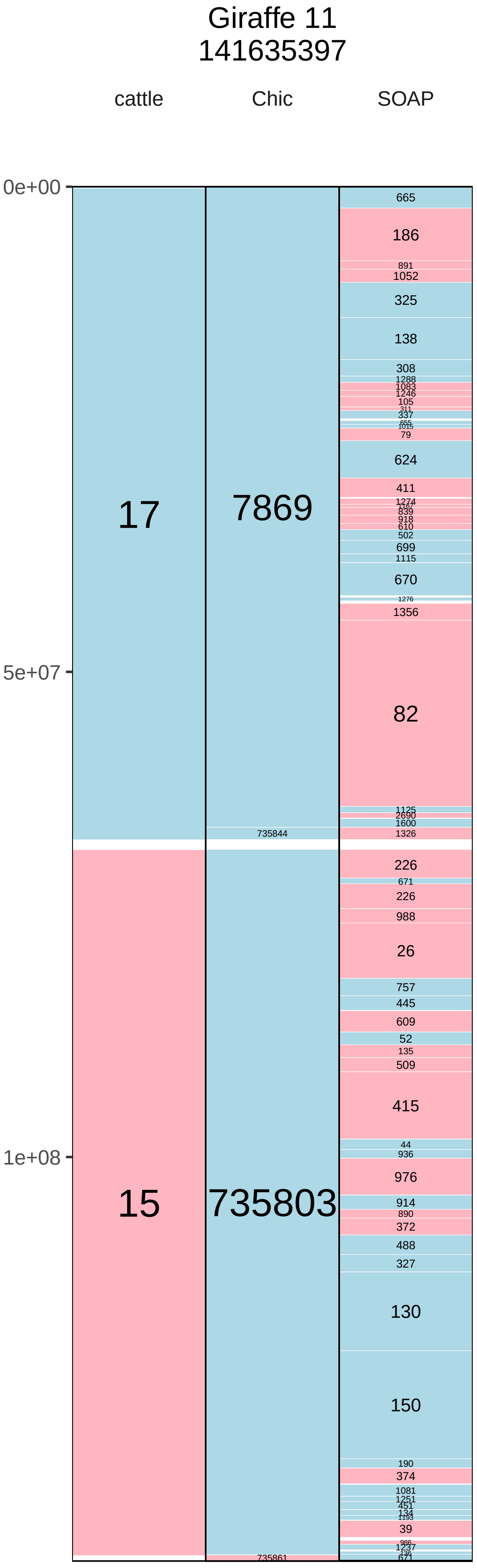

Giraffe 11
141635397
cattle
Chic
SOAP
0e+00
5e+07
1e+08
17
15
7869
735844
735803
735861
665
186
891
1052
325
138
308
1288
1083
1246
105
311
337
655
1015
79
624
411
1274
839
918
610
502
699
1115
670
1276
1356
82
1125
2690
1600
1326
226
671
226
988
26
757
445
609
52
135
509
415
44
936
976
914
890
372
488
327
130
150
190
374
1081
1251
451
134
1193
39
986
1237
671

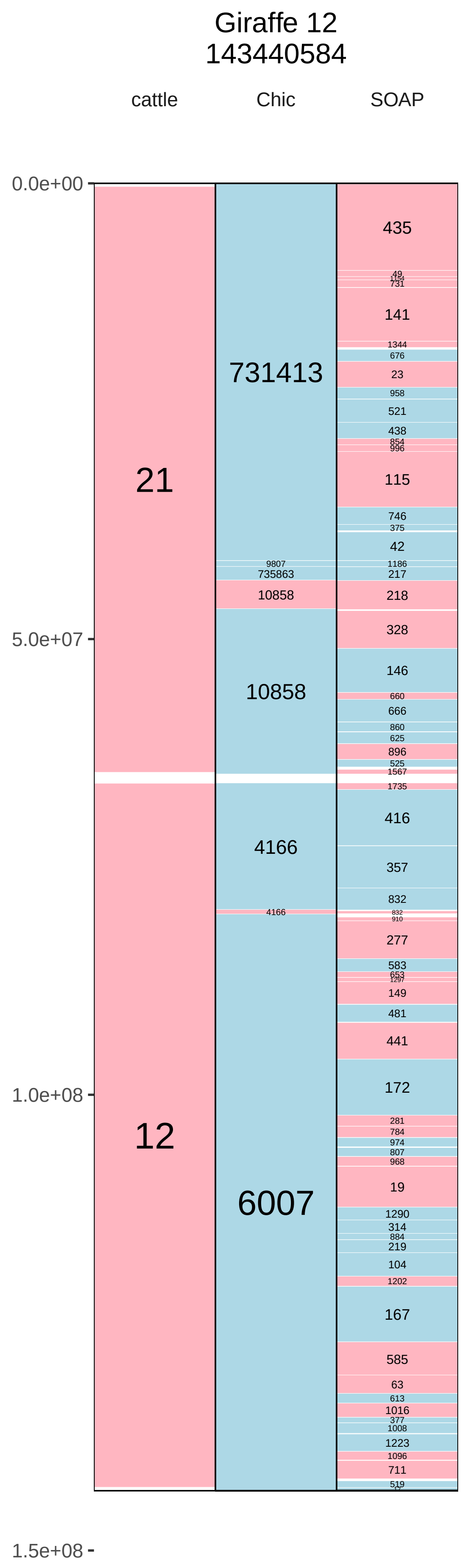
Giraffe 12
143440584
cattle
Chic
SOAP
0.0e+00
5.0e+07
1.0e+08
1.5e+08
21
12
731413
9807
735863
10858
10858
4166
4166
6007
435
141
23
115
42
218
328
146
666
896
416
357
832
277
149
481
441
172
19
104
167
585
63
711

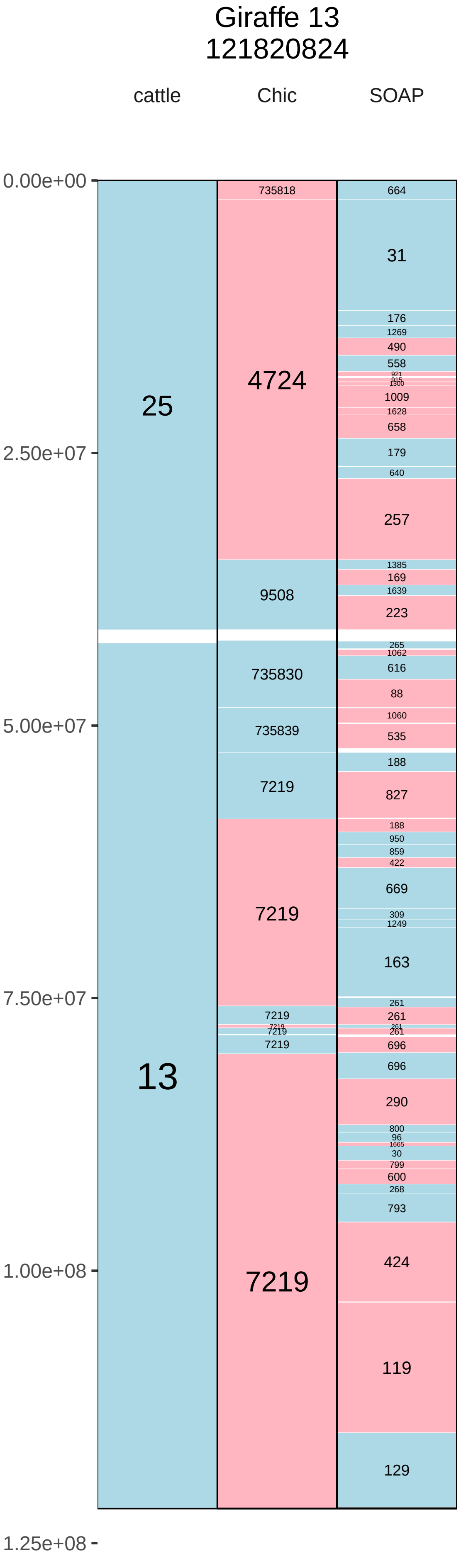
Giraffe 13
121820824
cattle
Chic
SOAP
0.00e+00
2.50e+07
5.00e+07
7.50e+07
1.00e+08
1.25e+08
25
13
735818
4724
9508
735830
735839
7219
7219
7219
7219
7219
7219
7219
664
31
176
1269
490
558
1009
1628
658
179
640
257
1385
169
1639
223
265
1062
616
88
1060
535
188
827
188
950
859
422
669
309
1249
163
261
261
261
696
696
290
800
96
1665
30
799
600
268
793
424
119
129

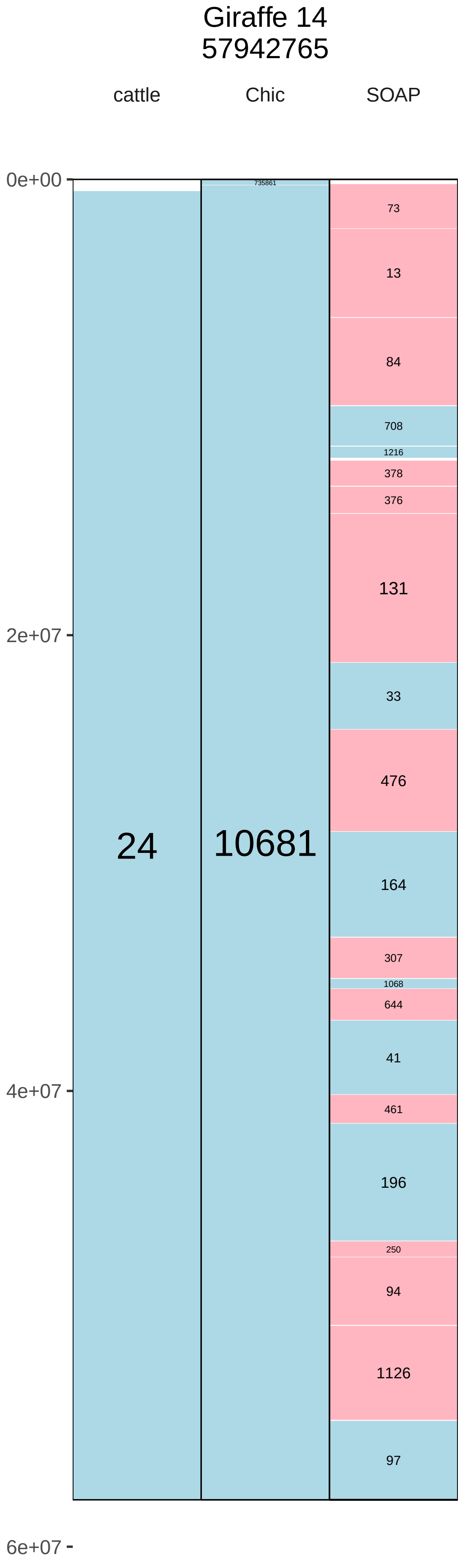
Giraffe 14
57942765
cattle
Chic
SOAP
0e+00
2e+07
4e+07
6e+07
24
10681
735861
73
13
84
708
1216
378
376
131
33
476
164
307
1068
644
41
461
196
250
94
1126
97

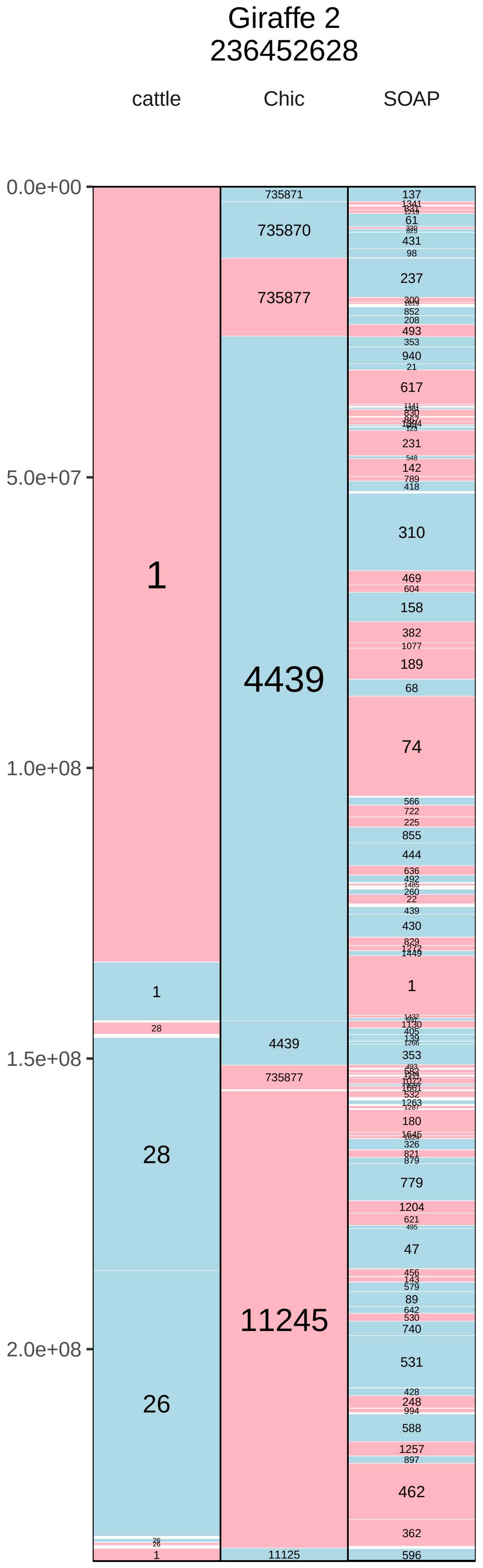
Giraffe 2
236452628
cattle
Chic
SOAP
0.0e+00
5.0e+07
1.0e+08
1.5e+08
2.0e+08
1
28
26
735871
735870
735877
4439
11245
11125
137
61
431
98
237
852
208
493
353
940
21
617
231
142
310
469
158
382
189
68
74
566
722
225
855
444
636
439
430
1
353
180
326
779
1204
621
47
89
740
531
248
588
1257
897
462
362
596

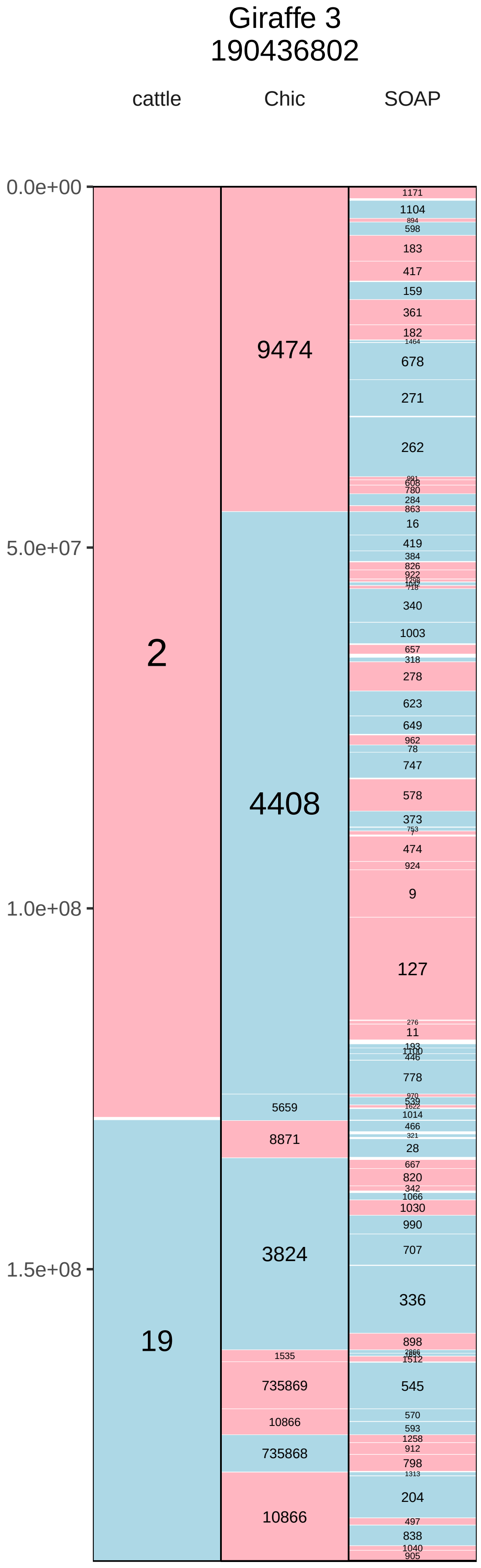
Giraffe 3
190436802
cattle
Chic
SOAP
0.0e+00
5.0e+07
1.0e+08
1.5e+08
2
19
9474
4408
5659
8871
3824
1535
735869
10866
735868
10866
1171
1104
598
183
417
159
361
182
678
271
262
780
284
863
16
419
384
826
340
1003
657
318
278
623
649
962
78
747
578
373
474
924
9
127
11
778
1014
466
28
667
820
342
1066
1030
990
707
336
898
1512
545
570
593
1258
912
798
1313
204
497
838
1040
905

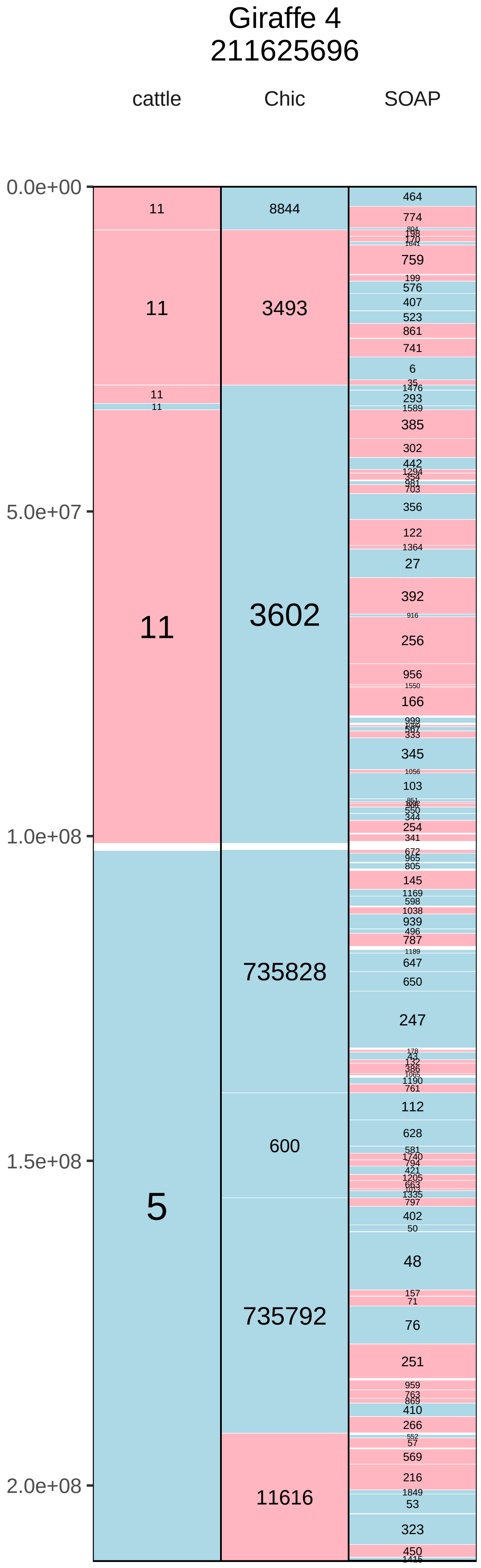

Giraffe 4
211625696
cattle
Chic
SOAP
0.0e+00
5.0e+07
1.0e+08
1.5e+08
2.0e+08

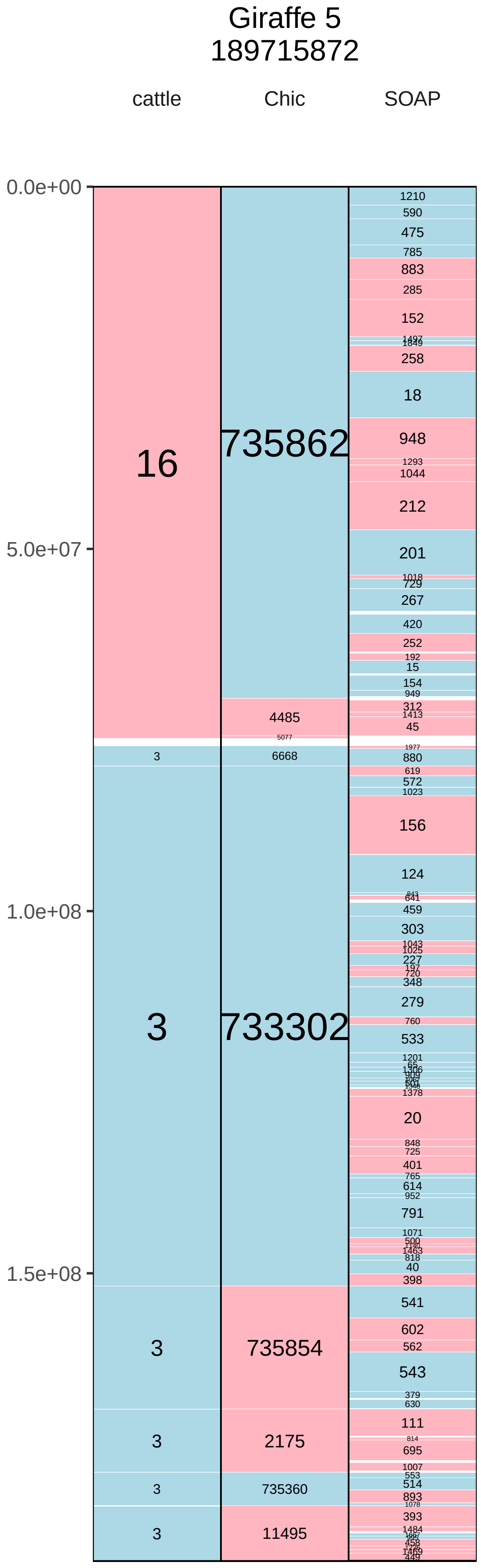

Giraffe 5
189715872
cattle
Chic
SOAP
0.0e+00
5.0e+07
1.0e+08
1.5e+08
16
735862
3
733302
735854
2175
735360
11495
4485
6668

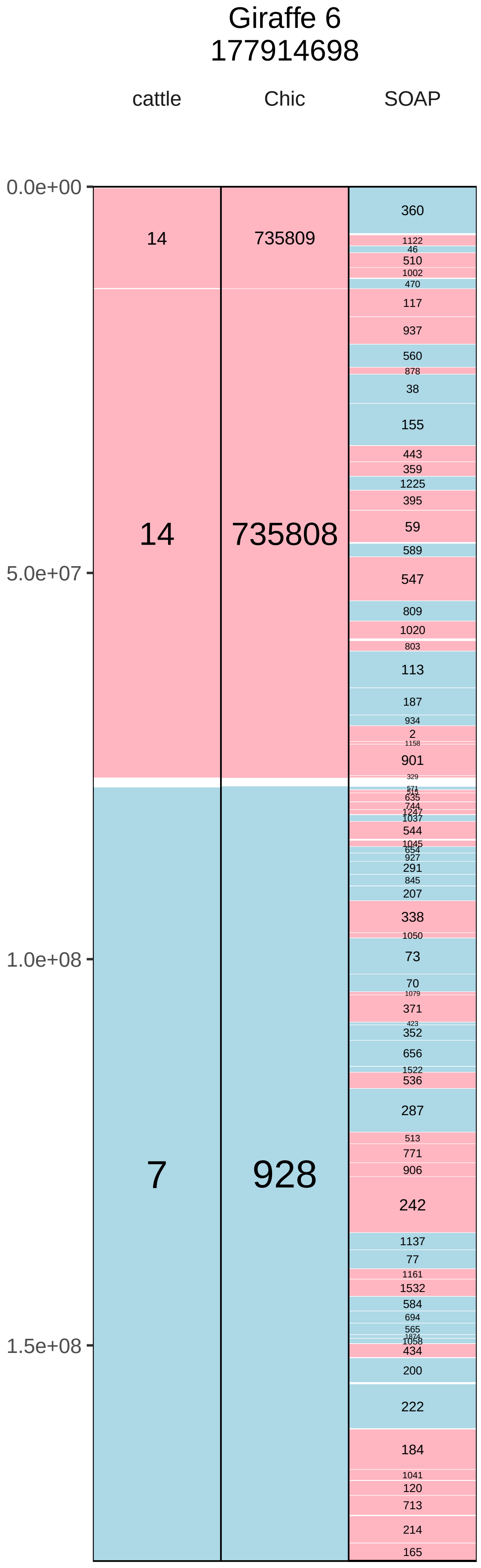

Giraffe 6
177914698
cattle
Chic
SOAP
0.0e+00
5.0e+07
1.0e+08
1.5e+08
14
735809
14
735808
7
928
360
1122
46
510
1002
470
117
937
560
878
38
155
443
359
1225
395
59
589
547
809
1020
803
113
187
934
2
1158
901
329
544
338
1050
73
70
1079
371
423
352
656
1522
536
287
513
771
906
242
1137
77
1161
1532
584
694
565
434
200
222
184
1041
120
713
214
165

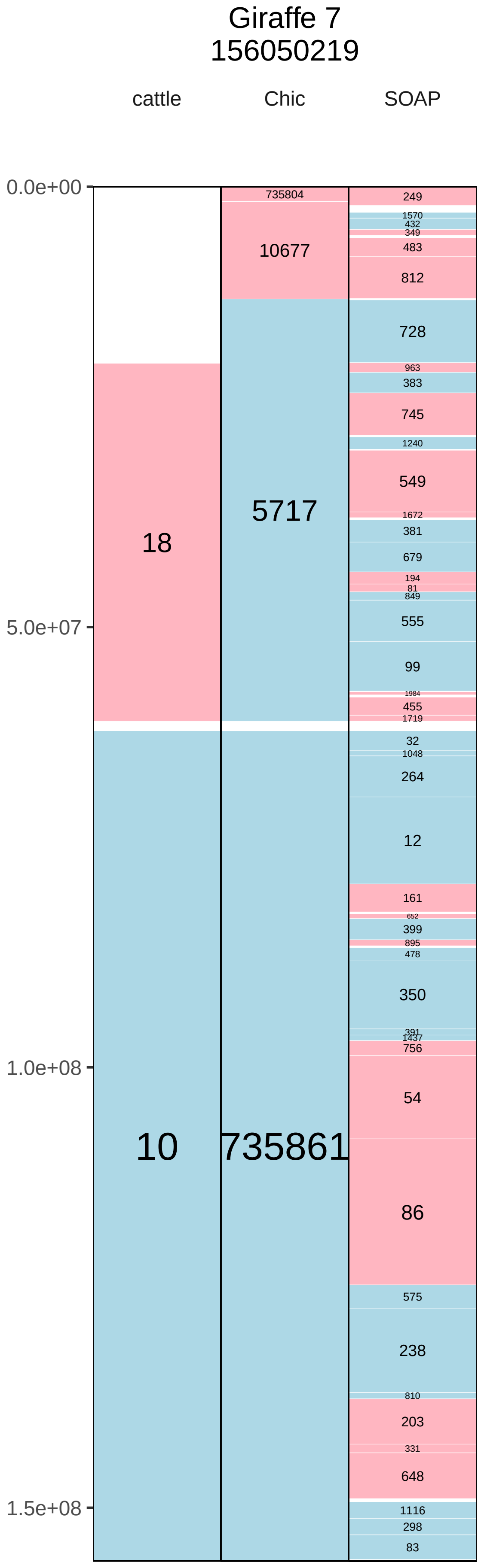

Giraffe 7
156050219
cattle
Chic
SOAP
0.0e+00
5.0e+07
1.0e+08
1.5e+08
18
10
735804
10677
5717
735861
249
1570
432
349
483
812
728
963
383
745
1240
549
1672
381
679
194
81
849
555
99
1984
455
1719
32
1048
264
12
161
652
399
895
478
350
391
1437
756
54
86
575
238
810
203
331
648
1116
298
83

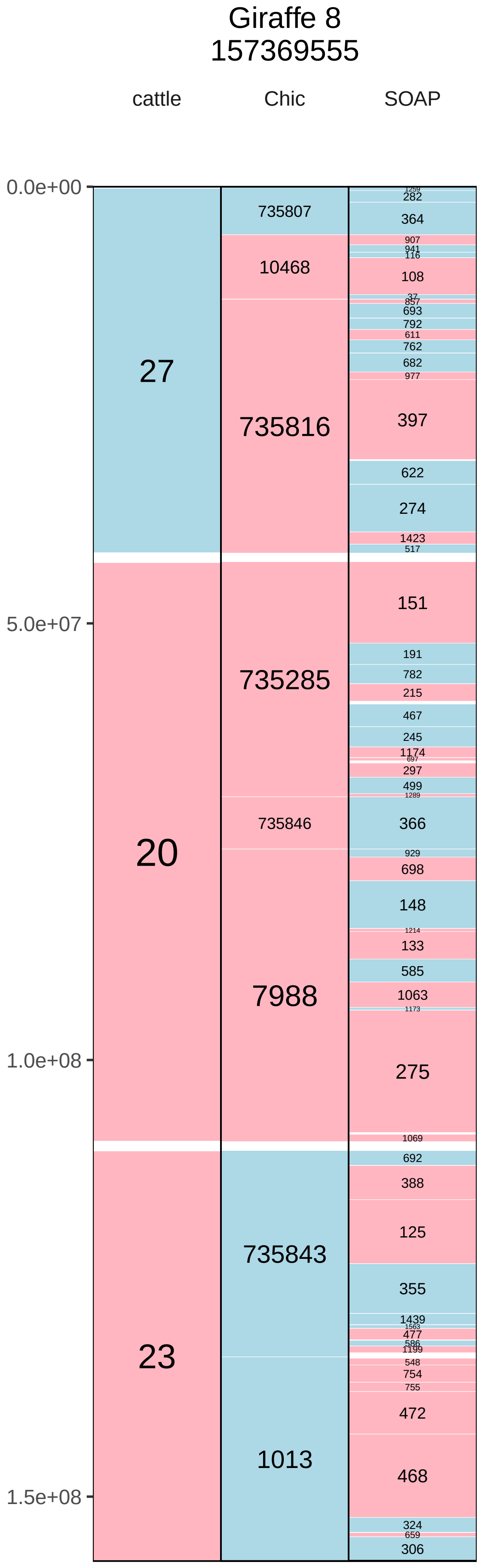
Giraffe 8
157369555
cattle
Chic
SOAP
0.0e+00
5.0e+07
1.0e+08
1.5e+08
27
20
23
735807
10468
735816
735285
735846
7988
735843
1013
282
364
907
941
116
108
857
693
792
611
762
682
977
397
622
274
1423
517
151
191
782
215
467
245
1174
297
499
366
929
698
148
133
585
1063
275
1069
692
388
125
355
1439
477
548
754
755
472
468
324
659
306

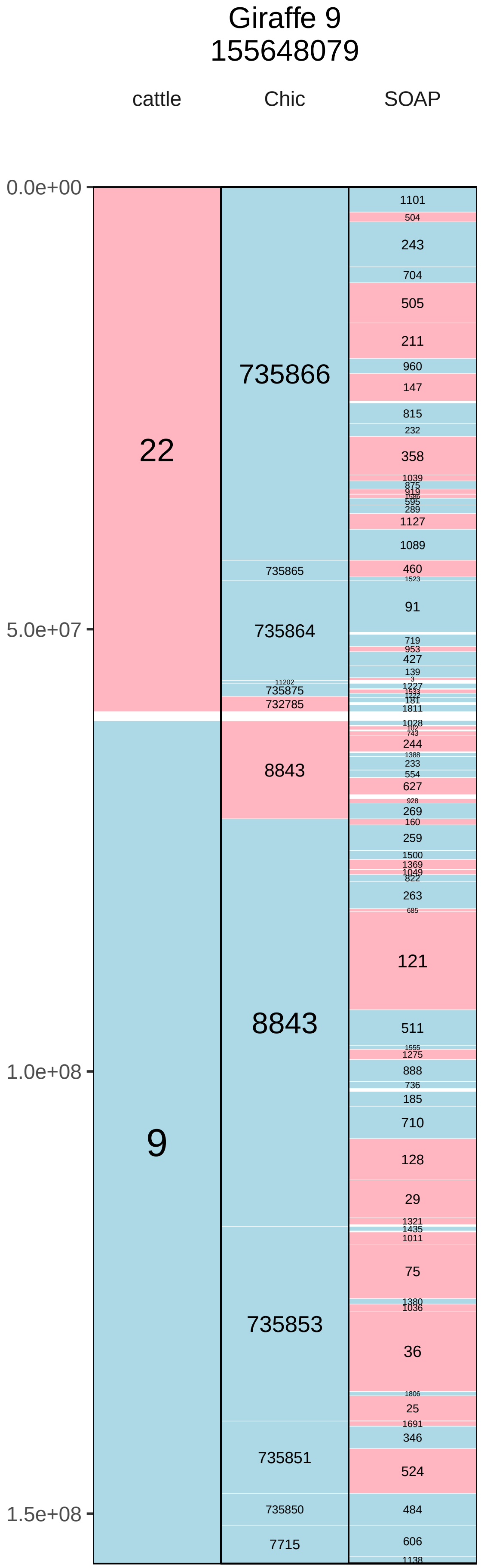

Giraffe 9
155648079
cattle
Chic
SOAP
0.0e+00
5.0e+07
1.0e+08
1.5e+08
22
9
735866
735865
735864
11202
735875
732785
8843
8843
735853
735851
735850
7715
1101
504
243
704
505
211
960
147
815
232
358
1039
875
595
289
1127
1089
460
1523
91
719
953
427
139
1227
181
1811
1028
743
244
1388
233
554
627
928
269
160
259
1500
1369
1049
822
263
685
121
511
1555
1275
888
736
185
710
128
29
1321
1435
1011
75
1380
1036
36
1806
25
1691
346
524
484
606
1138

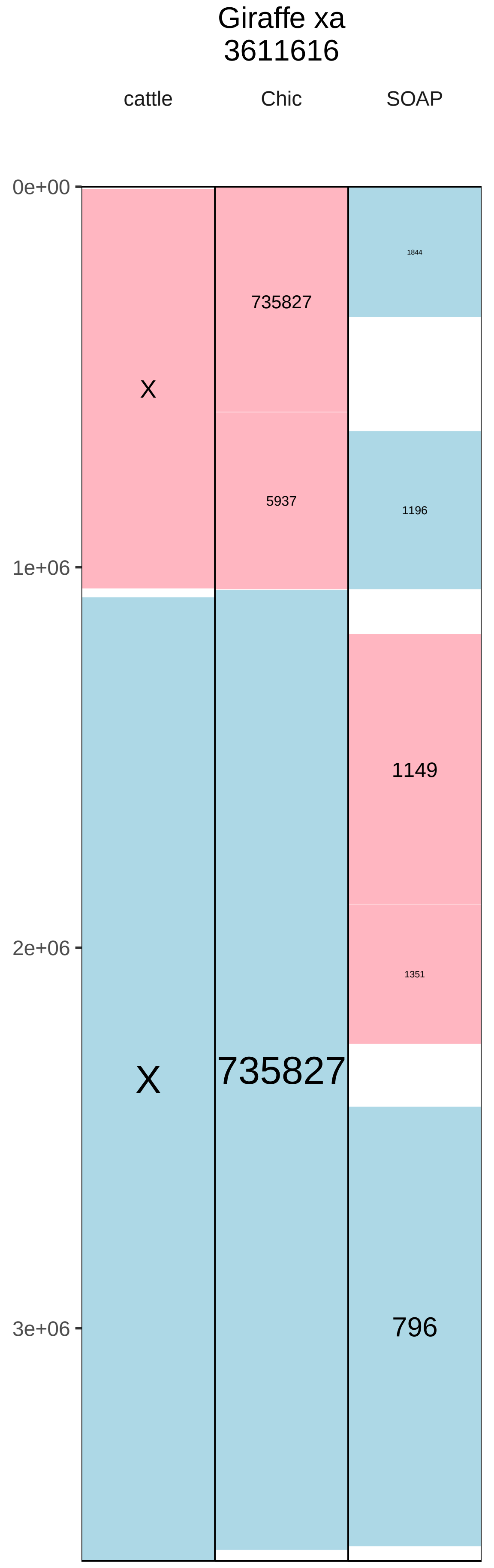

Giraffe xa
3611616
cattle
Chic
SOAP
0e+00
1e+06
2e+06
3e+06
X
X
735827
5937
735827
1844
1196
1149
1351
796

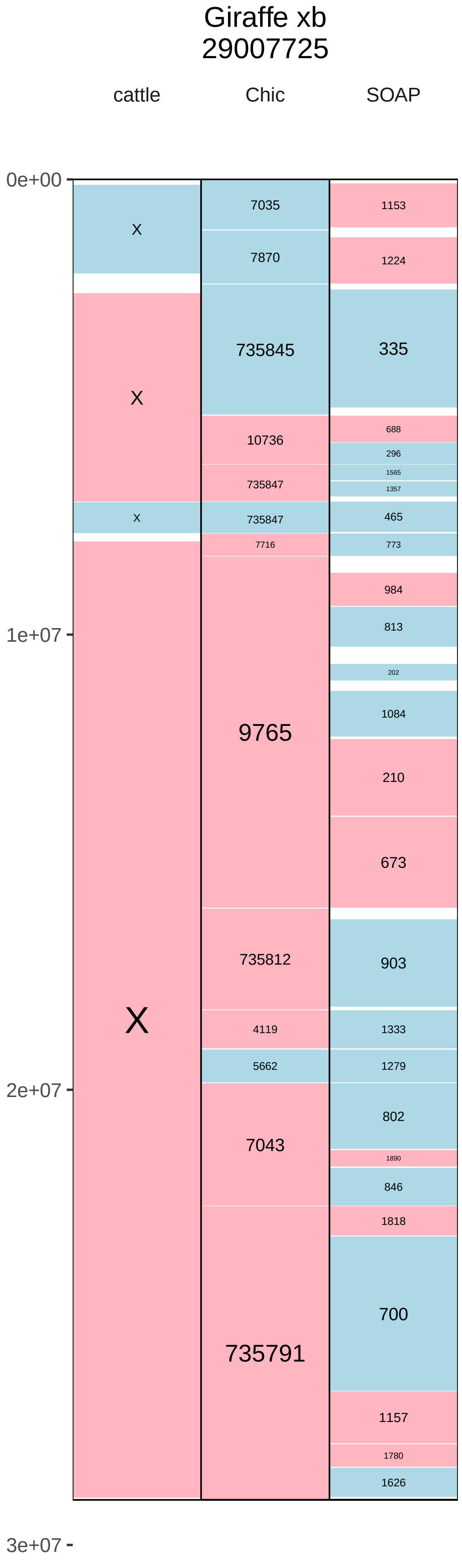

Giraffe xb
29007725
cattle
Chic
SOAP
0e+00
1e+07
2e+07
3e+07
X
X
X
X
7035
7870
735845
10736
735847
735847
7716
9765
735812
4119
5662
7043
735791
1153
1224
335
688
296
1565
1357
465
773
984
813
202
1084
210
673
903
1333
1279
802
1890
846
1818
700
1157
1780
1626

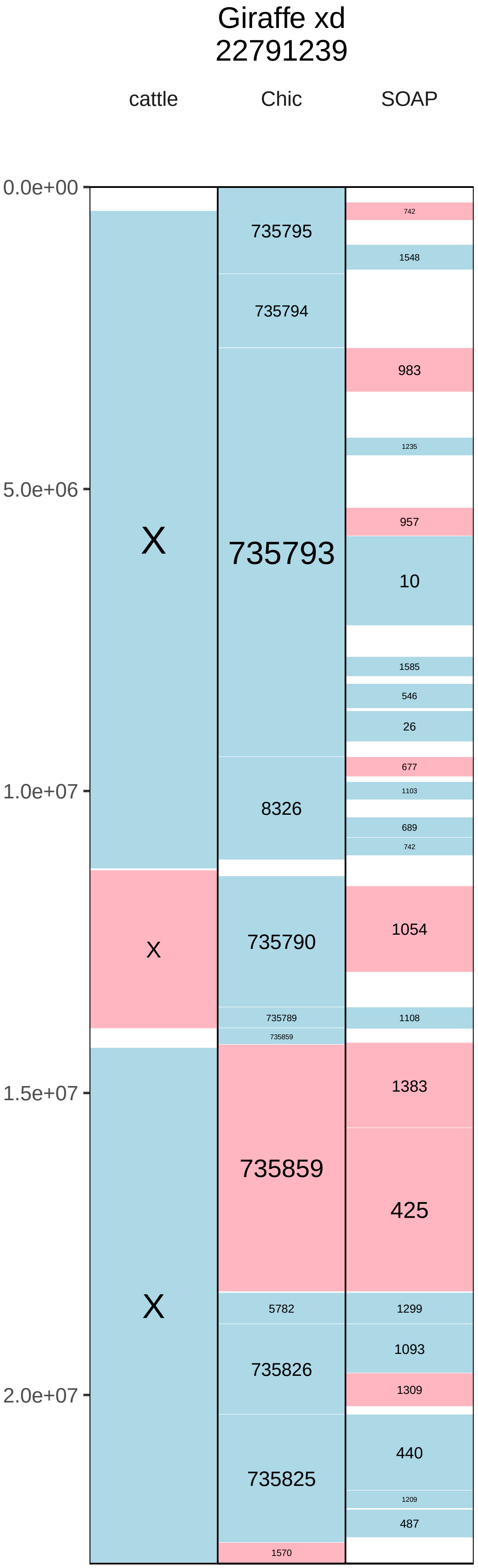
Giraffe xd
22791239
cattle
Chic
SOAP
0.0e+00
5.0e+06
1.0e+07
1.5e+07
2.0e+07
X
X
X
735795
735794
735793
8326
735790
735789
735859
735859
5782
735826
735825
1570
742
1548
983
1235
957
10
1585
546
26
677
1103
689
742
1054
1108
1383
425
1299
1093
1309
440
1209
487

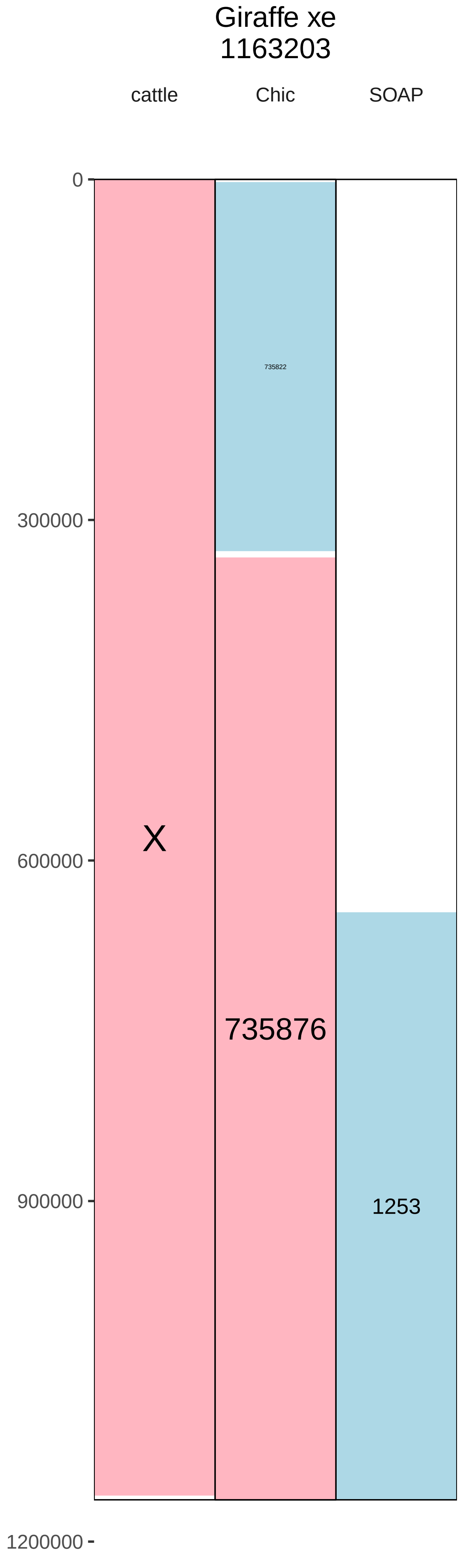
Giraffe xe
1163203
cattle
Chic
SOAP
0
300000
600000
900000
1200000
735822
X
735876
1253

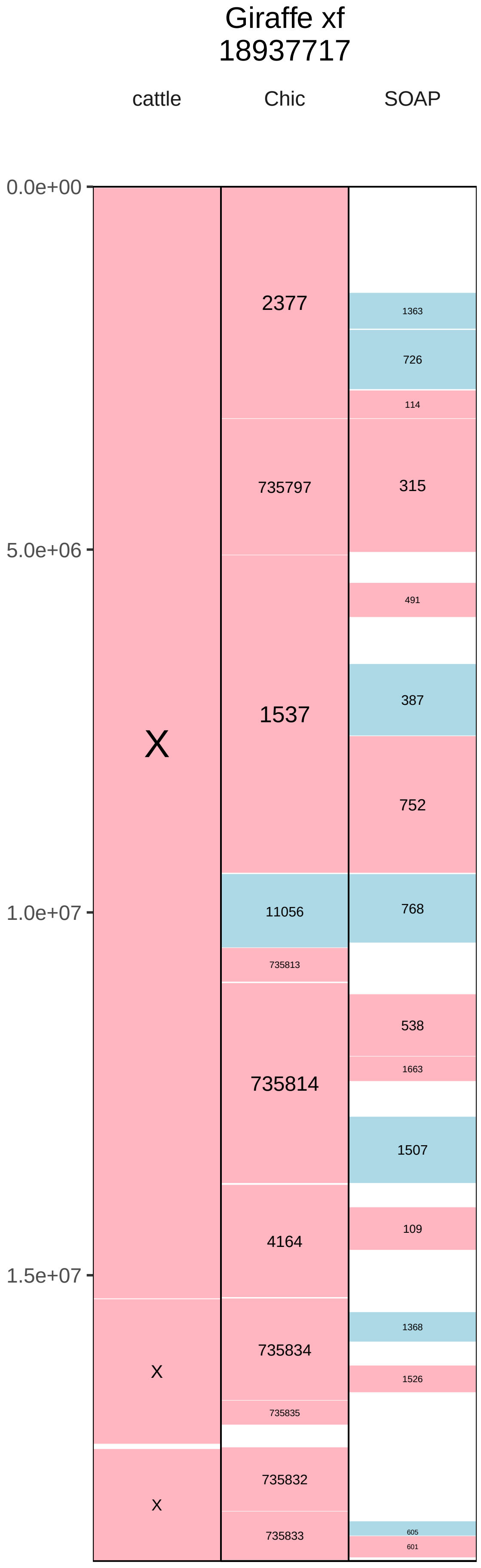
Giraffe xf
18937717
cattle
Chic
SOAP
0.0e+00
5.0e+06
1.0e+07
1.5e+07
X
x
x
2377
735797
1537
11056
735813
735814
4164
735834
735835
735832
735833
1363
726
114
315
491
387
752
768
538
1663
1507
109
1368
1526
605
601

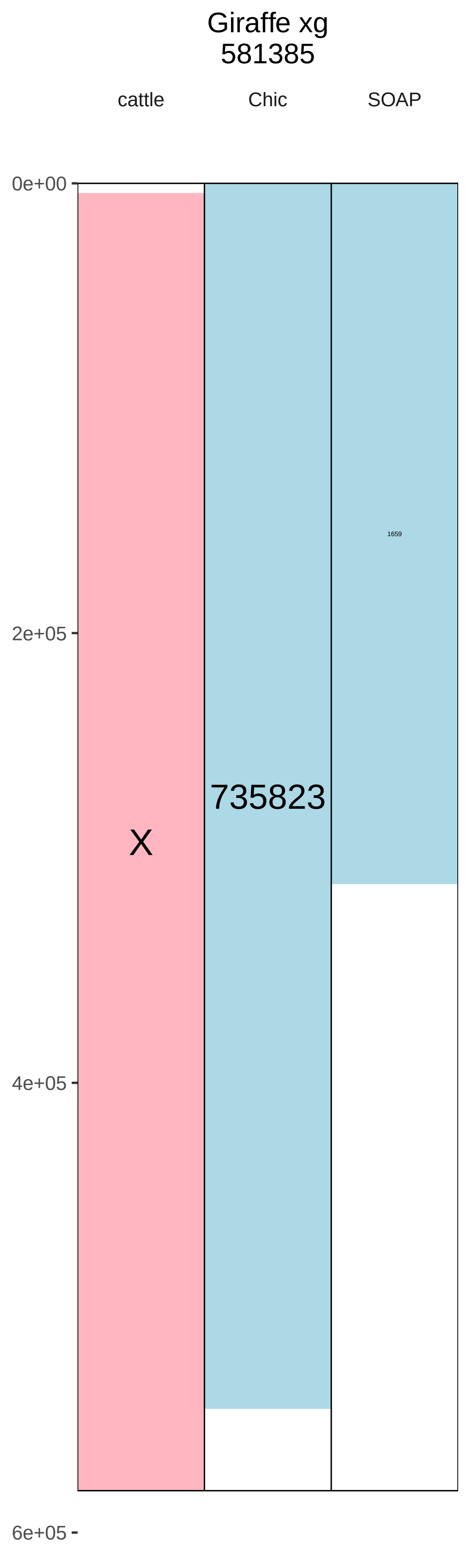

Giraffe xg
581385
cattle
Chic
SOAP
0e+00
2e+05
4e+05
6e+05
X
735823
1659

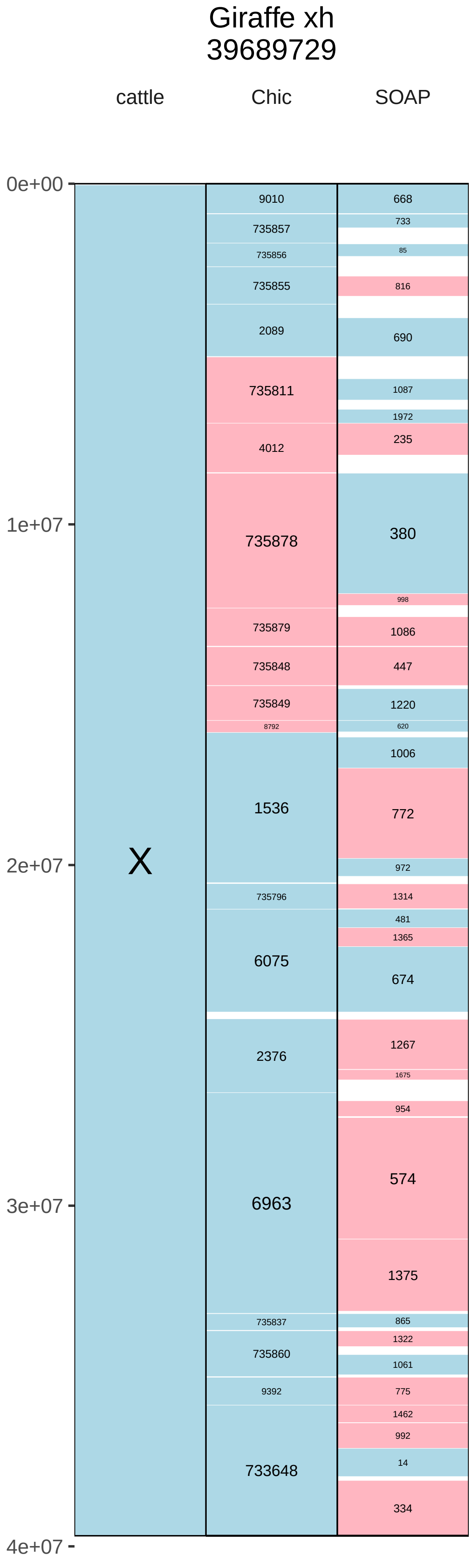

Giraffe xh
39689729
cattle
Chic
SOAP
0e+00
1e+07
2e+07
3e+07
4e+07
X
9010
735857
735856
735855
2089
735811
4012
735878
735879
735848
735849
8792
1536
735796
6075
2376
6963
735837
735860
9392
733648
668
733
85
816
690
1087
1972
235
380
998
1086
447
1220
620
1006
772
972
1314
481
1365
674
1267
1675
954
574
1375
865
1322
1061
775
1462
992
14
334

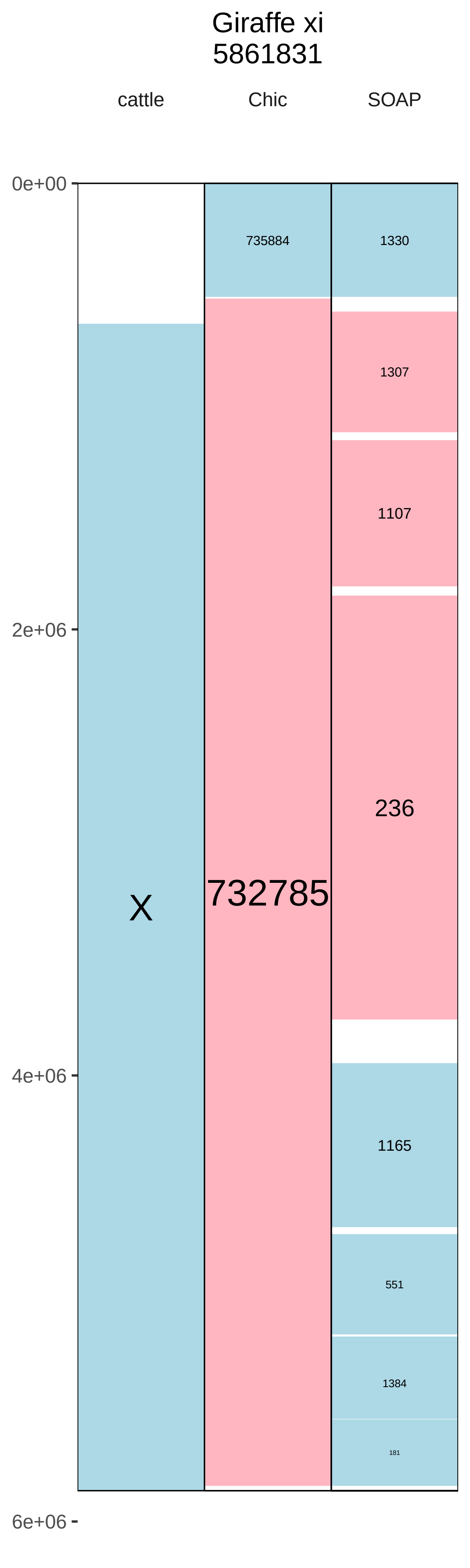
Giraffe xi
5861831
cattle
Chic
SOAP
0e+00
2e+06
4e+06
6e+06
735884
1330
1307
1107
236
732785
X
1165
551
1384
181

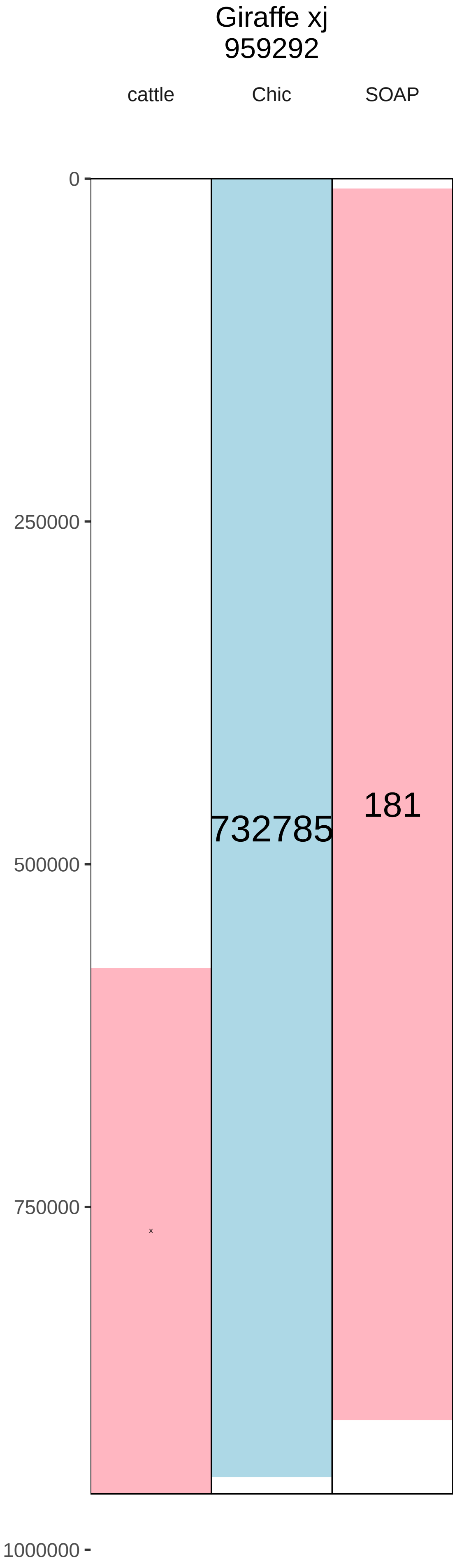

Giraffe xj
959292
cattle
Chic
SOAP
0
250000
500000
750000
1000000
732785
181
x
