# Supplementary material for: An integrated chromosome-scale genome assembly of the Masai giraffe (Giraffa camelopardalis tippelskirchi)
Source: Gigascience. 2019 Jul 30;8(8):giz090. doi: 10.1093/gigascience/giz090 (PMC6669057; doi:10.1093/gigascience/giz090)
Supplement: giz090_Response_to_Reviewer_Comments_Original_Submission [file giz090_response_to_reviewer_comments_original_submission.pdf]

Author's Response To Reviewer Comments

Close

Dear Mr Zauner,

We are very grateful for your time and the time of four referees who reviewed our manuscript. We were pleased to find that the paper could be accepted for publication in GigaScience just after some minor modifications.

As you pointed in your email, one of the main issues raised by most of the reviewers was to include a wider comparison with the publicly available giraffe genome assembly. We agree with all the reviewers that a more detailed comparison would be beneficial to illustrate the improvements gained by our new effort, however, we encountered several issues while trying to do so. The main one being the lack of publicly available gene annotations for the Agaba et al., 2016 assembly. This has impeded us to benchmark our gene annotation and assess the improvement. We addressed all the reviewer's comments and our detailed responses are included below.

We also submit the modified manuscript with all changes highlighted as well as the modified tables and supplementary files.

Sincerely yours,

Marta Farré
Denis Larkin
Harris Lewin

Reviewer reports:

Reviewer #1:
The manuscript is about resequencing the genome of the Masai Giraffe and constructing a high quality and contiguity chromosome-level reference assembly for the species. The goal was to improve over the previous assembly (Agaba et al. 2016), so that the resulting genome sequence would be applicable for evolutionary studies and species conservation. The authors used a well-selected set of cutting-edge next generation genomics technologies (short- and long-read sequencing; proximity ligation sequencing) and bioinformatics tools to achieve the goal. The manuscript is very well written and clear.
However, while reading the manuscript, I was very positive and enthusiastic in the beginning but disappointed in the end. To me, the manuscript looked like a well-written and detailed methodological manual about how to generate a high-quality chromosome-level annotated genome assembly for any mammalian species. I could recommend it for this purpose to any graduate student and postdoc. Disappointment was because I learned very little about the Masai Giraffe as such. Maybe this was the intention of the authors and in accordance with the profile of the journal. If so, this is a beautiful methodological paper on how to sequence, assemble and annotate mammalian genomes. If not, the authors should provide more information specific to the species.

Response: We thank the reviewer for his/her thoughtful comments. We fully agree with the reviewer that Giraffe is an iconic animal and that our chromosome-scale assembly will lead to new insights into its biology. However, we decided to publicly release the genome as soon as possible to allow the rest of the community access to this genome. Following GigaSicence Data Note style format, only a short description of the assembly should be included. As stated in GigaScience webpage: "One of the aims of a Data Note is to incentivize and more rapidly release data before subsequent detailed analysis has been carried out."

Specific comments:

Comment 1. Like any other re-sequencing project, the manuscript should provide a comparison with the previous assembly together with examples illustrating the improvement (filling gaps or improving gene models, etc.).

Response: In reality, our project was not a resequencing project. The primary sequencing and de novo assembly of short reads was completed well before the paper by Agaba was published. However, our goal was always to produce a chromosome-scale assembly for the giraffe, and this process took longer than expected. While we agree with the reviewer that a more comprehensive comparison with the previous assembly would be beneficial, gene annotations for the Agaba et al., 2016 version were not publicly released. As such, we were not able to compare gene models between both assemblies. To compare the contiguity of the assemblies, we performed a pair-wise alignment between the publicly available assembly and our assembly and identified the discordant scaffolds. We included a new supplementary table (Supplementary Table 5) reporting these scaffolds. As the reviewer will see, only 18 scaffolds from the Agaba assembly were split when aligned to our assembly, and 11,005 joins were introduced, showing that our assembly is more contiguous.

Comment 2. It would be worth mentioning that together with the study by Agaba et al., there are now sequences of 3 female Masai Giraffe genomes.

Response: We thank the reviewer for this suggestion. Although in Agaba et al., 2016 the authors mentioned a second giraffe assembly, only one was fully described in their Supplementary Table 2 and deposited in GenBank. Therefore, we do not feel confident saying that there are now three giraffe genome assemblies publicly available.

Comment 3. The authors mention in Introduction about the specific biological features and adaptations of the Masai Giraffe, but do not use the improved sequence assembly to show this. If genome-wide analysis of signatures of selection is a too big task (and it likely is), the authors should revisit the genes under selection as pointed out by Agaba et al. (2016), and demonstrate how the new assembly improves this information.

Response: As mentioned above, the primary purpose of this data note is to describe the details of the first chromosome-scale assembly for any giraffe. The genome-wide analysis for signatures of selection is beyond the scope of this article and will be considered elsewhere. We were not able to revisit the genes under selection indicated in the Agaba et al., 2016 publication because the authors did not provide the location of these genes nor the gene models from them, making it impossible to compare gene annotations.

Comment 4. The authors mention in Introduction about the use of the assembly for conservation efforts of the giraffe but do not show in the manuscript how it will be done.

Response: We thank the reviewer for their suggestions. We included a new section in the manuscript, entitled “Conclusions”. In this section, we give examples of cases where the availability of a genome assembly has fostered conservation genomics approaches.

Comment 5. Fig. 1: please specify whether this is the photo of the sequenced individual or just a representative of a species

Response: This photo is of a representative of the species and not the individual that was sequenced. We have clarified it in the figure legend. It now reads:
“Figure 1. A representative adult female Masai giraffe (Giraffa camelopardalis tippelskirchi) in the Masai Mara national park, Kenya. Picture taken by Bjørn Christian Tørrissen, licence CC BY-SA 3.0.”

Reviewer #2:
The paper sets out to give a chromosome-scale assembly for the Masai giraffe, which is achieved. The results presented give a de novo assembly and chromosomal analyses.
I have a few general comments and specific queries.

Comment 1. It would have been great to draw out a few unique observations about the genes specifically referring to giraffe.
The abstract mentions "many missing fragments and fragmented genes" when introducing the previously published giraffe genome. Which were these fragmented genes, and have they been

improved?
No comparison with previous genome apart from BUSCO numbers. Which areas were improved the most? Is everything else the same?
Which genes were missing before? Are they in repeat regions? Are they any of the adaptation genes mentioned in the previous assembly paper?

Response. We thank the reviewer for the suggestion. However, Agaba and colleagues did not make publicly available their gene annotations. Moreover, as reported in their publication, genes were annotated in the giraffe assembly using only lastZ: "We used gene annotations for assemblies of the cow and dog genomes to identify putative coding regions for giraffe and okapi, as follows. We downloaded gene models for the cow assembly called bosTau4 from Ensembl (www.ensembl.org). The union of all intervals annotated as coding, as well as 150 bp flanks, were used to extract sequence from the bosTau4 assembly, forming the mapping target. Giraffe reads were mapped to the target using lastz. Mapping required an alignment with at least 60% of the read length as matched bases, and at least 3 matches better than the second best alignment."

As such, we believe that our de novo and homology-based gene annotation is more reliable. More importantly, we included the GFT file with the annotation as part of this manuscript, making it accessible to all the researcher community.

Comment 2. I would have liked a summary figure of all of the chromosomes, as the data provided does not give this.

Response. A supplementary figure (Suppl. Fig 1) includes all the giraffe chromosomes compared to the cattle genome and with the placed SOAPdenovo and SOAPdenovo+Chicago scaffolds. One of these chromosomes can also be found in Figure 2. Moreover, in figure 2 the reviewer can find the giraffe karyotype showing the placement of BACs used to assemble the genome as well as a Circos plot comparing giraffe and cattle chromosomes.

Comment 3. Data analyses: Why hg19 and not GRCh38?
Genome annotation: Ensembl 64 used. Very old, almost 10 years old. Ens 64 is no longer supported in the browser. For GRCh37 why not use Ens 75?
Which version of SwissProt was used of the analysis?

Response. We agree with the reviewer that Ensembl 64 is an old version; however, the protein coding gene annotations in cattle, horse and mouse did not change significantly between Ensembl 64 and Ensembl 75, as shown in the table below. Only the number of protein coding genes annotated in the human genome increased. However, because we used a combination of de novo and homology-based annotations with four different species gene sets, we believe that our final merged gene set using GLEAN represents a comprehensive and reliable gene annotation set, containing similar number of genes than other ruminant genomes.

Table 1. Number of protein-coding genes in each species used for the homology-based step in Ensembl 64 and 75 versions.

Species	Ensembl 64	Ensembl 75	Difference
Cattle (UMD3.1)	24,616	24,621	5
Human (hg19)	21,181	22,827	1,646
Mouse (NCBIm38)	22,711	22,753	42
Horse (EquCab2)	20,436	20,449	13

Comment 4. Figure 3: I'd like a better legend explaining this. It's not very clear.

Response. We have expanded Figure 3 legend. It now reads:

"Figure 3. Benchmarking of genome completeness for the four giraffe assemblies using BUSCO. The BUSCO dataset of the mammalia_odb9 including 4,104 genes was used to assess the completeness of the four giraffe genome assemblies, as well as the previously published giraffe genome (ASM165123v1

[9]). The newly released cattle (ARS−UCD1.2, GCA_002263795.2) and goat (ARS1, GCA_001704415.1) assemblies are included for comparison.

Comment 5. Supp table 5: There are a few errors in this table.
Mean exon per gene. I am assuming this is an error as the numbers are in thousands of exons per gene?
CDS is in bp. Shouldn't that be in amino acids as it refers to protein?
"Final" row at end of table. Not clear why this number is or how it was derived.

Response. We thank the reviewer for pointing us to this error. As the reviewer suggested, it is indeed an error and we have corrected the table. We renamed the "Final" row to "GLEAN", as we used this tool to combine the de novo and the homology-based annotations.

Reviewer #3:
Summary: In this manuscript, Farre et al. detail the generation of a new reference for the Masai Giraffe using a combination of short read sequence data, Dovetail Hi-C and reference-guided scaffold correction. The assembly statistics, as presented, show higher degrees of scaffold continuity and BUSCO completeness than the previous Giraffe reference. It's highly likely that this assembly will be of use to the community and that Giraffe represents an interesting leaf in the Artiodactyla clade. Still, I found several areas where the manuscript did not provide enough context or details on the analysis.

Comment 1. Pg 5 Line 23: The details of the PCR chimera check need further fleshing out. Did the authors use genomic DNA as the template or sequencing libraries? Since not all SF joint boundaries were tested via PCR amplification, it would be helpful to supply a supplementary table showing which boundaries were tested. Finally, how was the 158X physical coverage threshold determined?

Response. We thank the reviewer for the suggestions. To clarify, PCRs were performed using genomic DNA as a template and the mapping of the sequencing libraries was used to establish the 158X physical coverage. A new supplementary table has been included indicating the scaffold ID, the PCR results and the read physical coverage. We have amended the text and it now reads:
"Chimerism was evaluated using PCR amplification of Masai giraffe DNA with primers that flank the RACA-defined split of SF joint boundaries (Supplementary Table 2 and Supplementary Table 3). Because we were only able to test 76% of the putatively chimeric SOAPdenovo scaffolds, we mapped short- and long-insert size read libraries to the SOAPdenovo assembly to establish a minimum physical coverage of reads that mapped across the SF joint intervals, following previous publications [18]. By comparing the PCR results and the read mapping coverage, we established 158x as the minimum physical coverage that allowed differentiation of scaffolds that were likely to be chimeric from those that were likely to be authentic (Supplementary Table 2).

Comment 2. Pg 7 Line 32: The fragmentary X chromosome assembly is only mentioned in the abstract, but it represents a major limitation of this assembly version. A reason why this chromosome was not successfully scaffolded should be listed here or in the previous sections.

Response. We agree with the reviewer that the fragmentary assembly of chromosome X might be an issue for some researchers. It is known that chromosome X in Cetartiodactyla species is highly rearranged (Proskuryakova et al., 2017), and therefore, using RACA in this clade would not improve much the contiguity in this chromosome. This fact, combined with the high fragmentation already present in the SOAPdenovo+Chicago assembly, where chromosome X was assembled into 66 scaffolds, while giraffe chromosome 3, of a similar size, was assembled into 19 scaffolds, explains why chromosome X is represented in 10 fragments in the final assembly. We have included this explanation in the manuscript, and it now reads: "The final genome assembly comprised PCFs placed on 14 giraffe autosomes and 10 chromosome X fragments (Table 1). Because chromosome X in Cetartiodactyls (including giraffe, cattle, and pigs) has been highly rearranged during evolution [19], tools such as RACA, that use a reference-assisted assembly approach, will have limited success in increasing the contiguity of the assembly of sex chromosomes in the Cetartiodactyl clade."

Comment 3. Table1: The listed assembly lengths vary considerably. It would be helpful to list the percentages of gap sequence in each assembly iteration.

Response. A new row in Table 1 has been added showing the percentage of gap sequence in each assembly.

Comment 4. Figure3: If one were to believe the BUSCO scores, the original assembly scaffolds (SOAPdenovo) were the "most complete" version of the assembly and subsequent scaffolding actually removed single copy genes from the assembly. This is a known issue with BUSCO evaluation, but it deserves mentioning in the results and discussion. Confirming that BUSCO single copy genes were deleted by RACA or Chicago edits would be important to report.

Response. As the reviewer pointed and it has been previously established, comparing different BUSCO runs on different versions of genome assemblies might produce inconsistent results (as shown by several issues reported in the BUSCO github page, f.e. https://gitlab.com/ezlab/busco/issues/94 and in Waterhouse et al., 2017). Only 34 BUSCO single copy genes found in the SOAPdenovo assembly were reported missing in the Final assembly. After looking at the responses from the authors in their github account, we re-ran BUSCO by modifying the hardcoded parameter of "maximum candidate region size of a contig" from 70 to 35% and found that all the inconsistencies disappeared. Since we modified the BUSCO code, we would prefer to not report these findings because they will not be reproducible by other researchers. As the reviewer suggested, we included an explanation of the discrepancies between the different genome assemblies in the main text. It now reads:
"Although comparing BUSCO results on different versions of genome assemblies might be inappropriate due to difference in parameter estimations [23], we found a high agreement between genome assemblies, with only 34 BUSCO single copy genes present in the SOAPdenovo assembly reported missing in the final assembly, while 42 BUSCO genes reported as fragmented and an additional 14 reported as missing in the SOAPdenovo assembly were labelled as complete in the final assembly. Overall, approximately 95% of the core mammalian gene set was complete in the SOAPdenovo and SOAPdenovo + Chicago assemblies; SOAPdenovo + RACA included 94% of the mammalian gene set, while the final chromosome-level assembly contained 95% complete BUSCO genes, similar to other reference-quality ruminant assemblies (94% for cattle ARS-UCD1.2 and goat ARS1)."

Comment 5. References: Citations to the manuscripts that accompanied the release of the cattle and goat reference genomes are missing.

Response. We have added the references to these manuscripts.

Reviewer #4:
The manuscript describes assembly of a genome of an important mammal, the giraffe, whose extraordinary physical features have interested evolutionary biologists for more than centuries. The assembly presented here fills the gaps that were previously unaddressed by Agaba et al and contribute significantly new resource to the analysis and understanding of mammalian evolution and specifically to giraffe biology.
The authors use a set of complementary sequencing approaches to generate huge amounts of sequence data, and combined these with appropriate sequence assembly tools to arrive at a reference genome of the giraffe that is comparable to those of cattle and goats (ARS1), furthermore chromosome evolution is ascertained with direct observation with FISH analysis. The paper is reasonably well written and organised, however, there are two sections that can be improved;

Comment 1. The section on genome evolution only highlights phylogenetic relationships of selected ruminants and can be perhaps bolstered by moving the results of synthetic comparison between cattle and giraffe to this section.

Response. Although we agree with the reviewer that a more in-depth analysis of the giraffe genome will lead to new insights into its biology, we decided to publicly release the genome as soon as possible to allow the rest of the community access it. As such, and following GigaSicence Data Note style format, only a short description of the assembly should be included. Even though we believe that synteny comparisons between giraffe and other species will provide insights into the biology of this and other species, in this particular work we used these comparisons to assess and assemble the genome at chromosome-level. Therefore, we would prefer to leave the paragraph about FISH and pair-wise alignment results in the assembly section.

Comment 2. The manuscript appears to end abruptly, and so I suggest that authors should consider adding a section with conclusion. Some of the elements for such a section are already in the manuscript

page 7 line 28 - 36.

Response. We thank the reviewer for this suggestion. We have included a new section in the manuscript, entitled “Conclusions”. It now reads:
“Herein, we report a de novo chromosome-scale genome assembly for Masai giraffe using a combination of sequencing and assembly methodologies aided by physical mapping of 153 BACs onto giraffe metaphase chromosomes. Gene and repeat annotation of the assembly identified a similar number of genes and transposable elements as found in other ruminant species. Following the example of the sable antelope [42] and the California condor [43], the new giraffe genome assembly will foster research into conservation of this charismatic species, serving as a foundation for characterizing the genetic diversity of wild and captive populations. Furthermore, the high quality, chromosome-scale assembly described in this report contributes to the goals of the Genome 10K Project [24] and the Earth BioGenome Project [25].”

Close
