# Supplementary material for: An integrated chromosome-scale genome assembly of the Masai giraffe (Giraffa camelopardalis tippelskirchi)
Source: Gigascience. 2019 Jul 30;8(8):giz090. doi: 10.1093/gigascience/giz090 (PMC6669057; doi:10.1093/gigascience/giz090)
Supplement: giz090_Response_to_Reviewer_Comments_Revision_1 [file giz090_response_to_reviewer_comments_revision_1.pdf]

Author's Response To Reviewer Comments

Close

Dear Mr Zauner,

We are pleased to know our manuscript has been accepted for publication in GigaScience. As requested, we have provided a clean version of the main text and added a note citing the recent publication in Science.

The text now reads:
"The underlying giraffe SOAPdenovo assembly described in this paper is the same as the one used by Chen and co-workers [45]."

Sincerely yours,

Marta Farré
Denis Larkin
Harris Lewin

Close
