# Supplementary material for: An integrated chromosome-scale genome assembly of the Masai giraffe (Giraffa camelopardalis tippelskirchi)
Source: Gigascience. 2019 Jul 30;8(8):giz090. doi: 10.1093/gigascience/giz090 (PMC6669057; doi:10.1093/gigascience/giz090)
Supplement: giz090_Reviewer_1_Report_Original_Submission — Terje Raudsepp -- 3/31/2019 Reviewed [file giz090_reviewer_1_report_original_submission.pdf]

Reviewer Report

Title: An integrated chromosome-scale genome assembly of the Masai Giraffe (Giraffa camelopardalis tippelskirchi)

Version: Original Submission **Date:** 3/31/2019

Reviewer name: Terje Raudsepp

Reviewer Comments to Author:

The manuscript is about resequencing the genome of the Masai Giraffe and constructing a high quality and contiguity chromosome-level reference assembly for the species. The goal was to improve over the previous assembly (Agaba et al. 2016), so that the resulting genome sequence would be applicable for evolutionary studies and species conservation. The authors used a well-selected set of cutting-edge next generation genomics technologies (short- and long-read sequencing; proximity ligation sequencing) and bioinformatics tools to achieve the goal. The manuscript is very well written and clear.
However, while reading the manuscript, I was very positive and enthusiastic in the beginning but disappointed in the end. To me, the manuscript looked like a well-written and detailed methodological manual about how to generate a high-quality chromosome-level annotated genome assembly for any mammalian species. I could recommend it for this purpose to any graduate student and postdoc. Disappointment was because I learned very little about the Masai Giraffe as such. Maybe this was the intention of the authors and in accordance with the profile of the journal. If so, this is a beautiful methodological paper on how to sequence, assemble and annotate mammalian genomes. If not, the authors should provide more information specific to the species.
Specific comments:
1. Like any other re-sequencing project, the manuscript should provide a comparison with the previous assembly together with examples illustrating the improvement (filling gaps or improving gene models, etc.).
2. It would be worth mentioning that together with the study by Agaba et al., there are now sequences of 3 female Masai Giraffe genomes.
3. The authors mention in Introduction about the specific biological features and adaptations of the Masai Giraffe, but do not use the improved sequence assembly to show this. If genome-wide analysis of signatures of selection is a too big task (and it likely is), the authors should revisit the genes under selection as pointed out by Agaba et al. (2016), and demonstrate how the new assembly improves this information.
4. The authors mention in Introduction about the use of the assembly for conservation efforts of the giraffe but do not show in the manuscript how it will be done.
5. Fig. 1: please specify whether this is the photo of the sequenced individual or just a representative of a species

Level of Interest

Please indicate how interesting you found the manuscript: Choose an item.

Quality of Written English

Please indicate the quality of language in the manuscript: Choose an item.

Declaration of Competing Interests

Please complete a declaration of competing interests, considering the following questions:

- Have you in the past five years received reimbursements, fees, funding, or salary from an organisation that may in any way gain or lose financially from the publication of this manuscript, either now or in the future?
- Do you hold any stocks or shares in an organisation that may in any way gain or lose financially from the publication of this manuscript, either now or in the future?
- Do you hold or are you currently applying for any patents relating to the content of the manuscript?
- Have you received reimbursements, fees, funding, or salary from an organization that holds or has applied for patents relating to the content of the manuscript?
- Do you have any other financial competing interests?
- Do you have any non-financial competing interests in relation to this paper?

If you can answer no to all of the above, write 'I declare that I have no competing interests' below. If your reply is yes to any, please give details below.

I declare that I have no competing interests' below

I agree to the open peer review policy of the journal. I understand that my name will be included on my report to the authors and, if the manuscript is accepted for publication, my named report including any attachments I upload will be posted on the website along with the authors' responses. I agree for my report to be made available under an Open Access Creative Commons CC-BY license (http://creativecommons.org/licenses/by/4.0/). I understand that any comments which I do not wish to be included in my named report can be included as confidential comments to the editors, which will not be published.

I agree to the open peer review policy of the journal

To further support our reviewers, we have joined with Publons, where you can gain additional credit to further highlight your hard work (see: https://publons.com/journal/530/gigascience). On publication of this paper, your review will be automatically added to Publons, you can then choose whether or not to claim your Publons credit. I understand this statement.

Yes Choose an item.
