# Supplementary material for: An integrated chromosome-scale genome assembly of the Masai giraffe (Giraffa camelopardalis tippelskirchi)
Source: Gigascience. 2019 Jul 30;8(8):giz090. doi: 10.1093/gigascience/giz090 (PMC6669057; doi:10.1093/gigascience/giz090)
Supplement: giz090_Reviewer_2_Report_Original_Submission — Jane Loveland -- 4/1/2019 Reviewed [file giz090_reviewer_2_report_original_submission.pdf]

Reviewer Report

Title: An integrated chromosome-scale genome assembly of the Masai Giraffe (Giraffa camelopardalis tippelskirchi)

Version: Original Submission **Date:** 4/1/2019

Reviewer name: Jane Loveland

Reviewer Comments to Author:

The paper sets out to give a chromosome-scale assembly for the Masai giraffe, which is achieved. The results presented give a de novo assembly and chromosomal analyses.
I have a few general comments and specific queries.
1. It would have been great to draw out a few unique observations about the genes specifically referring to giraffe.
The abstract mentions "many missing fragments and fragmented genes" when introducing the previously published giraffe genome. Which were these fragmented genes and have they been improved?
No comparison with previous genome apart from BUSCO numbers. Which areas were improved the most? Is everything else the same?
Which genes were missing before? Are they in repeat regions? Are they any of the adaptation genes mentioned in the previous assembly paper?
2. I would have liked a summary figure of all of the chromosomes, as the data provided does not give this.
3. Data analyses: Why hg19 and not GRCh38?
Genome annotation: Ensembl 64 used. Very old, almost 10 years old. Ens 64 is no longer supported in the browser. For GRCh37 why not use Ens 75?
Which version of SwissProt was used of the analysis?
4. Figure 3: I'd like a better legend explaining this. It's not very clear.
5. Supp table 5: There are a few errors in this table.
Mean exon per gene. I am assuming this is an error as the numbers are in thousands of exons per gene?
CDS is in bp. Shouldn't that be in amino acids as it refers to protein?
"Final" row at end of table. Not clear why this number is or how it was derived.

Level of Interest

Please indicate how interesting you found the manuscript: Choose an item.

Quality of Written English

Please indicate the quality of language in the manuscript: Choose an item.

Declaration of Competing Interests

Please complete a declaration of competing interests, considering the following questions:

- Have you in the past five years received reimbursements, fees, funding, or salary from an organisation that may in any way gain or lose financially from the publication of this manuscript, either now or in the future?
- Do you hold any stocks or shares in an organisation that may in any way gain or lose financially from the publication of this manuscript, either now or in the future?
- Do you hold or are you currently applying for any patents relating to the content of the manuscript?
- Have you received reimbursements, fees, funding, or salary from an organization that holds or has applied for patents relating to the content of the manuscript?
- Do you have any other financial competing interests?
- Do you have any non-financial competing interests in relation to this paper?

If you can answer no to all of the above, write 'I declare that I have no competing interests' below. If your reply is yes to any, please give details below.

I declare that I have no competing interests

I agree to the open peer review policy of the journal. I understand that my name will be included on my report to the authors and, if the manuscript is accepted for publication, my named report including any attachments I upload will be posted on the website along with the authors' responses. I agree for my report to be made available under an Open Access Creative Commons CC-BY license (http://creativecommons.org/licenses/by/4.0/). I understand that any comments which I do not wish to be included in my named report can be included as confidential comments to the editors, which will not be published.

I agree to the open peer review policy of the journal

To further support our reviewers, we have joined with Publons, where you can gain additional credit to further highlight your hard work (see: https://publons.com/journal/530/gigascience). On publication of this paper, your review will be automatically added to Publons, you can then choose whether or not to claim your Publons credit. I understand this statement.

Yes Choose an item.
