# Supplementary material for: An integrated chromosome-scale genome assembly of the Masai giraffe (Giraffa camelopardalis tippelskirchi)
Source: Gigascience. 2019 Jul 30;8(8):giz090. doi: 10.1093/gigascience/giz090 (PMC6669057; doi:10.1093/gigascience/giz090)
Supplement: giz090_Reviewer_3_Report_Original_Submission — Derek Bickhart -- 4/1/2019 Reviewed [file giz090_reviewer_3_report_original_submission.pdf]

Reviewer Report

Title: An integrated chromosome-scale genome assembly of the Masai Giraffe (Giraffa camelopardalis tippelskirchi)

Version: Original Submission **Date:** 4/1/2019

Reviewer name: Derek Bickhart

Reviewer Comments to Author:

Summary: In this manuscript, Farre et al. detail the generation of a new reference for the Masai Giraffe using a combination of short read sequence data, Dovetail Hi-C and reference-guided scaffold correction. The assembly statistics, as presented, show higher degrees of scaffold continuity and BUSCO completeness than the previous Giraffe reference. It's highly likely that this assembly will be of use to the community and that Giraffe represents an interesting leaf in the Artiodactyla clade. Still, I found several areas where the manuscript did not provide enough context or details on the analysis.

Pg 5 Line 23: The details of the PCR chimera check need further fleshing out. Did the authors use genomic DNA as the template or sequencing libraries? Since not all SF joint boundaries were tested via PCR amplification, it would be helpful to supply a supplementary table showing which boundaries were tested. Finally, how was the 158X physical coverage threshold determined?

Pg 7 Line 32: The fragmentary X chromosome assembly is only mentioned in the abstract but it represents a major limitation of this assembly version. A reason why this chromosome was not successfully scaffolded should be listed here or in the previous sections.

Table1: The listed assembly lengths vary considerably. It would be helpful to list the percentages of gap sequence in each assembly iteration.

Figure3: If one were to believe the BUSCO scores, the original assembly scaffolds (SOAPdenovo) were the "most complete" version of the assembly and subsequent scaffolding actually removed single copy genes from the assembly. This is a known issue with BUSCO evaluation, but it deserves mentioning in the results and discussion. Confirming that BUSCO single copy genes were deleted by RACA or Chicago edits would be important to report.

References: Citations to the manuscripts that accompanied the release of the cattle and goat reference genomes are missing.

Level of Interest

Please indicate how interesting you found the manuscript: Choose an item.

Quality of Written English

Please indicate the quality of language in the manuscript: Choose an item.

Declaration of Competing Interests

Please complete a declaration of competing interests, considering the following questions:

- Have you in the past five years received reimbursements, fees, funding, or salary from an organisation that may in any way gain or lose financially from the publication of this manuscript, either now or in the future?
- Do you hold any stocks or shares in an organisation that may in any way gain or lose financially from the publication of this manuscript, either now or in the future?
- Do you hold or are you currently applying for any patents relating to the content of the manuscript?
- Have you received reimbursements, fees, funding, or salary from an organization that holds or has applied for patents relating to the content of the manuscript?
- Do you have any other financial competing interests?
- Do you have any non-financial competing interests in relation to this paper?

If you can answer no to all of the above, write 'I declare that I have no competing interests' below. If your reply is yes to any, please give details below.

I declare that I have no competing interests

I agree to the open peer review policy of the journal. I understand that my name will be included on my report to the authors and, if the manuscript is accepted for publication, my named report including any attachments I upload will be posted on the website along with the authors' responses. I agree for my report to be made available under an Open Access Creative Commons CC-BY license (http://creativecommons.org/licenses/by/4.0/). I understand that any comments which I do not wish to be included in my named report can be included as confidential comments to the editors, which will not be published.

I agree to the open peer review policy of the journal

To further support our reviewers, we have joined with Publons, where you can gain additional credit to further highlight your hard work (see: https://publons.com/journal/530/gigascience). On publication of this paper, your review will be automatically added to Publons, you can then choose whether or not to claim your Publons credit. I understand this statement.

Yes Choose an item.
