# Supplementary material for: An integrated chromosome-scale genome assembly of the Masai giraffe (Giraffa camelopardalis tippelskirchi)
Source: Gigascience. 2019 Jul 30;8(8):giz090. doi: 10.1093/gigascience/giz090 (PMC6669057; doi:10.1093/gigascience/giz090)
Supplement: giz090_Supplemental_Files [file giz090_supplemental_files.zip › giraffe_SupplData_reviewerComments_DL.docx]

**Supplementary Data**

**Supplementary Table 1. Summary of sequenced libraries for *Giraffa camelopardalis tippelskirchi*.**

| **Library Type** | **Inset size (bp)** | **Read length (bp)** | **Total data (Gbp)** | **Sequence coverage (fold)** |
| --- | --- | --- | --- | --- |
| Illumina paired-end | 170 | 100_100 | 38.32 | 15.33 |
|  | 250 | 150_150 | 42.22 | 16.89 |
|  | 500 | 100_100 | 29.51 | 11.80 |
|  | 800 | 100_100 | 23.73 | 9.49 |
| Illumina  mate-pair | 2,000 | 50_50 | 19.98 | 7.99 |
|  | 5,000 | 50_50 | 7.68 | 3.07 |
|  | 10,000 | 50_50 | 6.29 | 2.52 |
|  | 20,000 | 50_50 | 3.37 | 1.35 |
| Illumina | Total | -- | 171.09 | 68.44 |
| Chicago | NA | 150_150 | 114.60 | 88.41 |

**Supplementary Table 2. Statistics for the scaffold split regions tested by PCR.**

| Statistics | Masai giraffe |
| --- | --- |
| Paired-end read physical coverage within tested scaffolds | 1-470X |
| No. RACA-split SF adjacencies (default parameters) | 41 |
| No. RACA-split SF adjacencies tested by PCR | 32 (100%) |
| No. amplified (confirmed adjacencies) | 13 (41%) |
| No. not amplified (adjacencies not confirmed^1^) | 19 (59%) |
| No. RACA-suggested SF adjacencies tested by PCR | 17 |
| No. amplified adjacencies (chimeric scaffold adjacencies) | 14 |
| No. not-amplified (adjacencies not confirmed) | 3 |
| Selected physical coverage threshold for stage 2 RACA^2^ | 158X |
| No. PCR tested RACA-split SF adjacencies below selected threshold^3^ | 20 (100%) |
| No. chimeric SF adjacencies | 12 (60%) |
| No. confirmed SF adjacencies | 5 (25%) |
| No. ambiguous SF adjacencies | 3 (15%) |
| No. PCR tested RACA-split SF adjacencies above selected threshold^3^ | 12 (100%) |
| No. chimeric SF adjacencies | 4 (33%) |
| No. confirmed SF adjacencies | 8 (67%) |
| No. ambiguous SF adjacencies | 0 (0%) |
| No. non-tested SF split adjacencies | 9 |

^1^ May include adjacencies from chimeric scaffolds or result from PCR failure.

^2^ Threshold was set from PCR results.

^3^ Subset of 32 RACA-split SF adjacencies tested by PCR.

**Supplementary Table 3. Putatively chimeric SOAPdenovo scaffolds.** PCR results and read physical coverage are reported for each SOAPdenovo scaffold flagged as putatively chimeric by RACA.

| **SOAPdenovo scaffold ID** | **Break start** | **Break end** | **Break size** | **PCR result** | **Read physical coverage** |
| --- | --- | --- | --- | --- | --- |
| 1849 | 674,057 | 674,336 | 279 | chimeric | 20 |
| 261 | 786,464 | 788,030 | 1,566 | chimeric | 27 |
| 747 | 3,559,144 | 3,559,221 | 77 | chimeric | 30 |
| 386 | 168,250 | 168,517 | 267 | chimeric | 47 |
| 605 | 250,192 | 250,893 | 701 | chimeric | 48 |
| 504 | 1,374,766 | 1,374,912 | 146 | chimeric | 56 |
| 82 | 19,214,561 | 19,216,651 | 2,090 | chimeric | 74 |
| 73 | 4,678,184 | 4,678,186 | 2 | chimeric | 102 |
| 1227 | 673,858 | 675,703 | 1,845 | chimeric | 109 |
| 26 | 5,657,006 | 5,657,203 | 197 | chimeric | 124 |
| 530 | 403,887 | 404,596 | 709 | chimeric | 144 |
| 745 | 241,330 | 243,559 | 2,229 | chimeric | 157 |
| 384 | 1,395,571 | 1,398,973 | 3,402 | chimeric | 250 |
| 696 | 1,649,904 | 1,649,906 | 2 | chimeric | 250 |
| 267 | 291,828 | 292,292 | 464 | chimeric | 275 |
| 181 | 259,811 | 262,955 | 3,144 | chimeric | 335 |
| 129 | 6,736,694 | 6,736,694 | 1 | not chimeric | 1 |
| 139 | 1,040,961 | 1,044,543 | 3,582 | not chimeric | 44 |
| 1023 | 1,165,571 | 1,169,364 | 3,793 | not chimeric | 68 |
| 1254 | 174,587 | 177,605 | 3,018 | not chimeric | 69 |
| 613 | 1,861,854 | 1,864,483 | 2,629 | not chimeric | 73 |
| 344 | 238,603 | 239,301 | 698 | not chimeric | 158 |
| 849 | 669,112 | 670,550 | 1,438 | not chimeric | 161 |
| 74 | 6,932,931 | 6,932,933 | 2 | not chimeric | 177 |
| 74 | 6,742,119 | 6,743,162 | 1,043 | not chimeric | 177 |
| 548 | 467,725 | 467,922 | 197 | not chimeric | 303 |
| 598 | 1,537,006 | 1,537,012 | 6 | not chimeric | 370 |
| 353 | 3,573,954 | 3,573,986 | 32 | not chimeric | 442 |
| 493 | 481,418 | 481,455 | 37 | not chimeric | 470 |
| 671 | 679,286 | 927,110 | 247,824 | not tested | 7 |
| 906 | 422,217 | 443,996 | 21,779 | not tested | 17 |
| 742 | 303,924 | 391,570 | 87,646 | not tested | 55 |
| 188 | 949,203 | 1,101,747 | 152,544 | not tested | 62 |
| 832 | 2,187,049 | 2,279,827 | 92,778 | not tested | 71 |
| 813 | 975,884 | 1,120,904 | 145,020 | not tested | 86 |
| 481 | 532,942 | 534,131 | 1,189 | not tested | 100 |
| 816 | 598,155 | 604,469 | 6,314 | not tested | 126 |
| 388 | 3,931,215 | 3,932,982 | 1,767 | not tested | 225 |
| 526 | 705,361 | 705,361 | 1 | unknown | 0 |
| 226 | 2,496,350 | 2,496,350 | 1 | unknown | 1 |
| 585 | 2,621,152 | 2,621,312 | 160 | unknown | 34 |

**Supplementary Table 4. Weighted average matrix to calculate scores for joints introduced by RACA.**

| Type of support | Weighted score | Value | Condition |
| --- | --- | --- | --- |
| FISH | 40% | 1 | At least two BACs following the same order and orientation |
|  |  | 0 | No BAC data |
|  |  | -1 | Disagreement with FISH data |
| Chicago | 30% | 1 | RACA and Chicago agree |
|  |  | 0 | RACA breaks the Chicago scaffold |
| Physical read coverage | 20% | 0-1 | Numerical coverage of read mapping |
| Syntenic information | 10% | 0-1 | Support from RACA |

**Supplementary Table 5.** **Coordinates of scaffolds from Agaba et al., 2016 split in the final assembly.**

| **Giraffe chromosome** | **Start** | **End** | **Agaba scaffold ID** | **Start** | **End** | **Orientation** |
| --- | --- | --- | --- | --- | --- | --- |
| 2 | 46839692 | 47068670 | LVKQ01S000277.1 | 211241 | 443380 | -1 |
| 4 | 11071804 | 11280761 | LVKQ01S000277.1 | 0 | 210460 | 1 |
| 4 | 21273410 | 21481110 | LVKQ01S001388.1 | 365283 | 574019 | 1 |
| 9 | 122762093 | 123113169 | LVKQ01S001388.1 | 0 | 363220 | 1 |
| 3 | 24941737 | 25015592 | LVKQ01S001899.1 | 397885 | 474332 | -1 |
| 13 | 11849724 | 12248680 | LVKQ01S001899.1 | 1265 | 395394 | 1 |
| 7 | 132935353 | 133259394 | LVKQ01S002080.1 | 3281 | 310613 | 1 |
| 12 | 91647969 | 92169698 | LVKQ01S002080.1 | 319561 | 845417 | 1 |
| 12 | 43653656 | 43719092 | LVKQ01S002548.1 | 0 | 65542 | -1 |
| 12 | 46788212 | 46865328 | LVKQ01S002548.1 | 66485 | 145871 | 1 |
| 1 | 183063015 | 183211599 | LVKQ01S002775.1 | 4929 | 152995 | -1 |
| 8 | 148468335 | 148547978 | LVKQ01S002775.1 | 154874 | 234604 | 1 |
| 1 | 194808077 | 194865171 | LVKQ01S002971.1 | 555 | 57944 | -1 |
| 10 | 122740264 | 122958160 | LVKQ01S002971.1 | 60741 | 282694 | -1 |
| xb | 6889708 | 7085022 | LVKQ01S003033.1 | 100929 | 299670 | -1 |
| xb | 7681661 | 7781152 | LVKQ01S003033.1 | 949 | 100929 | 1 |
| 4 | 7268630 | 7518861 | LVKQ01S004013.1 | 79082 | 337022 | -1 |
| 7 | 12031148 | 12105191 | LVKQ01S004013.1 | 0 | 77125 | -1 |
| 2 | 25801382 | 25857012 | LVKQ01S005965.1 | 0 | 56945 | -1 |
| 2 | 150942819 | 151144719 | LVKQ01S005965.1 | 56945 | 257953 | -1 |
| 2 | 149596562 | 149949096 | LVKQ01S006524.1 | 266967 | 606467 | 1 |
| 4 | 159892268 | 160153265 | LVKQ01S006524.1 | 0 | 262693 | -1 |
| 2 | 97937329 | 98127620 | LVKQ01S006753.1 | 51341 | 253739 | -1 |
| 2 | 98127719 | 98441061 | LVKQ01S006753.1 | 253739 | 572731 | 1 |
| xa | 2215254 | 2269754 | LVKQ01S006761.1 | 0 | 56811 | 1 |
| xa | 2295189 | 2401911 | LVKQ01S006761.1 | 64033 | 168924 | -1 |
| xa | 2409471 | 2532768 | LVKQ01S006761.1 | 192356 | 318682 | 1 |
| 9 | 55304679 | 55805628 | LVKQ01S009588.1 | 6258 | 476879 | 1 |
| 9 | 55903540 | 56597286 | LVKQ01S009588.1 | 478312 | 1138307 | 1 |
| 2 | 25701373 | 25801283 | LVKQ01S010455.1 | 292200 | 397527 | -1 |
| 2 | 151144818 | 151433826 | LVKQ01S010455.1 | 0 | 292200 | -1 |
| 10 | 158584646 | 158770154 | LVKQ01S010885.1 | 0 | 186112 | 1 |
| 10 | 160288184 | 160341600 | LVKQ01S010885.1 | 191714 | 246518 | -1 |
| 9 | 48643654 | 48857362 | LVKQ01S011345.1 | 0 | 219504 | 1 |
| 14 | 38856991 | 38989886 | LVKQ01S011345.1 | 218642 | 370666 | 1 |
| 11 | 71279262 | 71366634 | LVKQ01S011903.1 | 0 | 88699 | 1 |
| 11 | 71907984 | 72079741 | LVKQ01S011903.1 | 88699 | 264553 | 1 |

**Supplementary Table 6. Summary statics of interspersed** **repeat regions in *Giraffa camelopardalis tippelskirchi.***

| **TE type** | **RepBase TEs** | | **TE proteins** | | **Combined TEs** | |
| --- | --- | --- | --- | --- | --- | --- |
|  | **Length (Mbp)** | **% genome** | **Length (Mbp)** | **% genome** | **Length (Mbp)** | **% genome** |
| DNA | 63,049,324 | 2.47 | 6,358,796 | 0.25 | 63,440,093 | 2.49 |
| LINE | 592,905,912 | 23.24 | 360,103,044 | 14.11 | 612,470,369 | 24.00 |
| LTR | 124,159,048 | 4.87 | 9,042,458 | 0.35 | 125,417,171 | 4.92 |
| SINE | 240,843,867 | 9.44 | - | - | 240,843,867 | 9.44 |
| Other | 1,727 | 0.03 | - | - | 1,727 | 0.03 |
| Unknown | 883,041 | 6.77 | - | - | 883,041 | 6.77 |
| Total | 1,003,680,269 | 39.34 | 375,443,513 | 14.71 | 1,015,465,850 | 39.80 |

**Supplementary Table 7. General statistics of predicted protein-coding genes in *Giraffa camelopardalis tippelskirchi.***

| **Gene set** | | **Number** | **Single exon genes** | **Average transcript length**  **(bp)** | **Average CDS length (AAs)** | **Average exons per gene** | **Average exon length (bp)** | **Average intron length (bp)** |
| --- | --- | --- | --- | --- | --- | --- | --- | --- |
| De novo | Augustus | 25,015 | 4,940 | 16,898 | 1,195 | 6.56 | 182 | 2,826 |
|  | Genscan | 40,318 | 2,784 | 39,863 | 1,296 | 7.87 | 165 | 5,611 |
|  | SNAP | 65,015 | 6,394 | 52,492 | 637 | 4.82 | 132 | 13,557 |
| Homolog | human | 20,913 | 4,854 | 24,180 | 1,327 | 7.66 | 173 | 3,434 |
|  | mouse | 23,057 | 8,231 | 21,641 | 1,256 | 7.11 | 177 | 3,334 |
|  | cow | 22,674 | 7,554 | 19,882 | 1,195 | 7.12 | 168 | 3,055 |
|  | horse | 32,829 | 12,249 | 21,905 | 1,170 | 6.65 | 176 | 3,672 |
| Glean | | 21,621 | 3,007 | 41,695 | 1,524 | 8.54 | 178 | 5,327 |

**Supplementary Table 8. Summary statistics of gene function annotation.**

|  |  | **Number** | **Percentage (%)** |
| --- | --- | --- | --- |
| Total |  | 21,621 | 100.00 |
| Annotated | Swissprot | 18,910 | 87.46 |
|  | KEGG | 9,087 | 42.03 |
|  | InterPro | 16,137 | 74.64 |
|  | GO | 12,263 | 56.72 |
| Unannotated |  | 2,666 | 12.33 |

**Supplementary Figure 1.** Giraffe chromosomes showing homologous synteny blocks (HSBs) between giraffe and cattle. SOAPdenovo and SOAPdenovo + Chicago scaffolds are also displayed. Blue blocks indicate positive (+) orientation of tracks compared with the giraffe chromosome while red blocks, negative (−) orientation. Numbers inside each block represent cattle chromosomes or giraffe scaffold IDs.
